# Impact of *Escherichia coli* K12 and O18:K1 on human platelets: Differential effects on platelet activation, RNAs and proteins

**DOI:** 10.1038/s41598-018-34473-w

**Published:** 2018-11-01

**Authors:** A. V. Fejes, M. G. Best, W. A. van der Heijden, A. Vancura, H. Verschueren, Q. de Mast, T. Wurdinger, C. Mannhalter

**Affiliations:** 1 Department of Laboratory Medicine, Medical University, Vienna, 1090 Austria; 2 Department of Neurosurgery, Cancer Center Amsterdam, Amsterdam UMC, Vrije Universiteit Amsterdam, Amsterdam, 1081 HV The Netherlands; 3 Department of Pathology, Cancer Center Amsterdam, Amsterdam UMC, Vrije Universiteit Amsterdam, Amsterdam, 1081 HV The Netherlands; 4 Brain Tumour Center Amsterdam, Amsterdam UMC, Vrije Universiteit Amsterdam, Amsterdam, 1081 HV The Netherlands; 5 Department of Internal Medicine, Radboud University Medical Center, Nijmegen, 6500 HB The Netherlands

## Abstract

Blood platelets can interact with bacteria, possibly leading to platelet activation, cytokine and microparticle release and immune signalling. Besides, bacteria can also affect the platelet RNA content. We investigated the impact of non-pathogenic K12 and pathogenic O18:K1 *Escherichia (E.) coli* strains on platelet activation, RNA expression patterns, and selected proteins. Depending on bacteria concentration, contact of platelets with *E. coli* K12 lead to an increase of P-selectin (24–51.3%), CD63 (15.9–24.3%), PAC-1 (3.8–14.9%) and bound fibrinogen (22.4–39%) on the surface. *E. coli* O18:K1 did not affect these markers. Sequencing analysis of total RNA showed that *E. coli* K12 caused a significant concentration change of 103 spliced mRNAs, of which 74 decreased. For the RNAs of *HMBS* (logFC = +5.73), *ATP2C1* (logFC = −3.13) and *LRCH4* (logFC = −4.07) changes were detectable by thromboSeq and Tuxedo pipelines. By Western blot we observed the conversion of HMBS protein from a 47 kDA to 40 kDa product by *E. coli* K12, O18:K1 and by purified lipopolysaccharide. While ATP2C1 protein was released from platelets, *E. coli* either reduced the secretion or broke down the released protein making it undetectable by antibodies. Our results demonstrate that different *E. coli* strains influence activation, RNA and protein levels differently which may affect platelet-bacteria crosstalk.

## Introduction

Blood platelets are anucleated cells, derived from megakaryocytes in the bone marrow. They are continuously shed into and cleared from the blood stream maintaining a high abundance with 150–400 × 10^9^ cells per litre of whole blood.

Platelets have an important role in haemostasis and thrombosis. They are packed with organelles, granules, RNA and proteins, which they primarily receive from their precursor cells. They have the capability to sequester proteins and RNA while in circulation^1,2^. It has been shown that platelets have a rich repertoire of RNAs, including ribosomal, circular and micro RNAs^3–5^, and ~9500 messenger RNAs^6^. Platelets contain a functional cellular machinery and have the capability to splice pre-mRNA into its mature form^7^. It has been shown that activation of splicing can be induced by lipopolysaccharide (LPS)^8^, thrombin^9^ or a septic environment^10^. Furthermore, cancer^11,12^ and cardiovascular diseases^13^ can influence the platelet RNA profile. Upon thrombin activation, translation of certain spliced RNAs to proteins has been reported^14,15^, which proved the presence of a translational machinery in platelets.

Besides haemostasis, platelets are important for humoral as well as cellular immune responses. They are able to interact with bacteria, which may result in their activation, aggregation, release of granules and platelet-leukocyte complex formation^16–19^. Recently it has been shown that platelets can act as cellular scavengers; they collect deposited bacteria and recruit phagocytes to boost the inflammatory reaction^20^. The interaction of platelets with Gram-positive bacteria, such as *Staphylococcus aureus* and *Streptococcus sanguinis*, has been extensively studied. It has been shown that both strains are able to trigger platelet aggregation^21,22^, *S. sanguinis* can induce cytokine release from platelets^23^, whereas *S. aureus* enhances thrombocytopenia^24^. The crosstalk between platelets and Gram-negative bacteria is less well characterized, although it has been shown that Gram-negative *Escherichia coli*^25,26^, *Helicobacter pylori*^27–30^ and *Klebsiella pneumoniae*^31–33^ have an activating or - in some cases - aggregating effect on platelets. *E. coli* are commensal bacteria of the gastrointestinal tract in humans and rarely cause disease. However, clones with specific virulence attributes exist which are able to induce clinical syndromes such as enteric disease, urinary tract infections and sepsis^34^. Some *E. coli* strains interact with platelets via the LPS ligand TLR4^35^, FcγRIIA or integrin complex αIIbβ3^36,37^. Little is known about the molecular consequences of the interactions between platelets and *E. coli*, and how they influence RNA or protein expression patterns or release of platelet contents.

We investigated the effects of a non-pathogenic (K12) and a pathogenic (O18:K1) *E. coli* strain on human platelets. We found that contact with *E. coli* K12 increases the activation markers P-selectin and CD63 on the platelet surface as well as PAC-1 and fibrinogen binding, while the pathogenic *E. coli* O18:K1 did not affect these markers. By next generation RNA sequencing, we found that the two *E. coli* strains affected different spliced platelet RNAs (mRNAs). Using two bioinformatics pipelines for analysis of RNA fingerprints we identified significant effects of *E. coli* on the mRNAs *HMBS*, *ATP2C1* and *LRCH4*. To see whether these three proteins were present in platelets and were influenced by the bacteria, we analysed HMBS, ATP2C1 and LRCH4 in platelet lysates and releasates by Western blot and ELISA.

## Results

### *E. coli* K12 affects platelet activation

**Figure 1:**
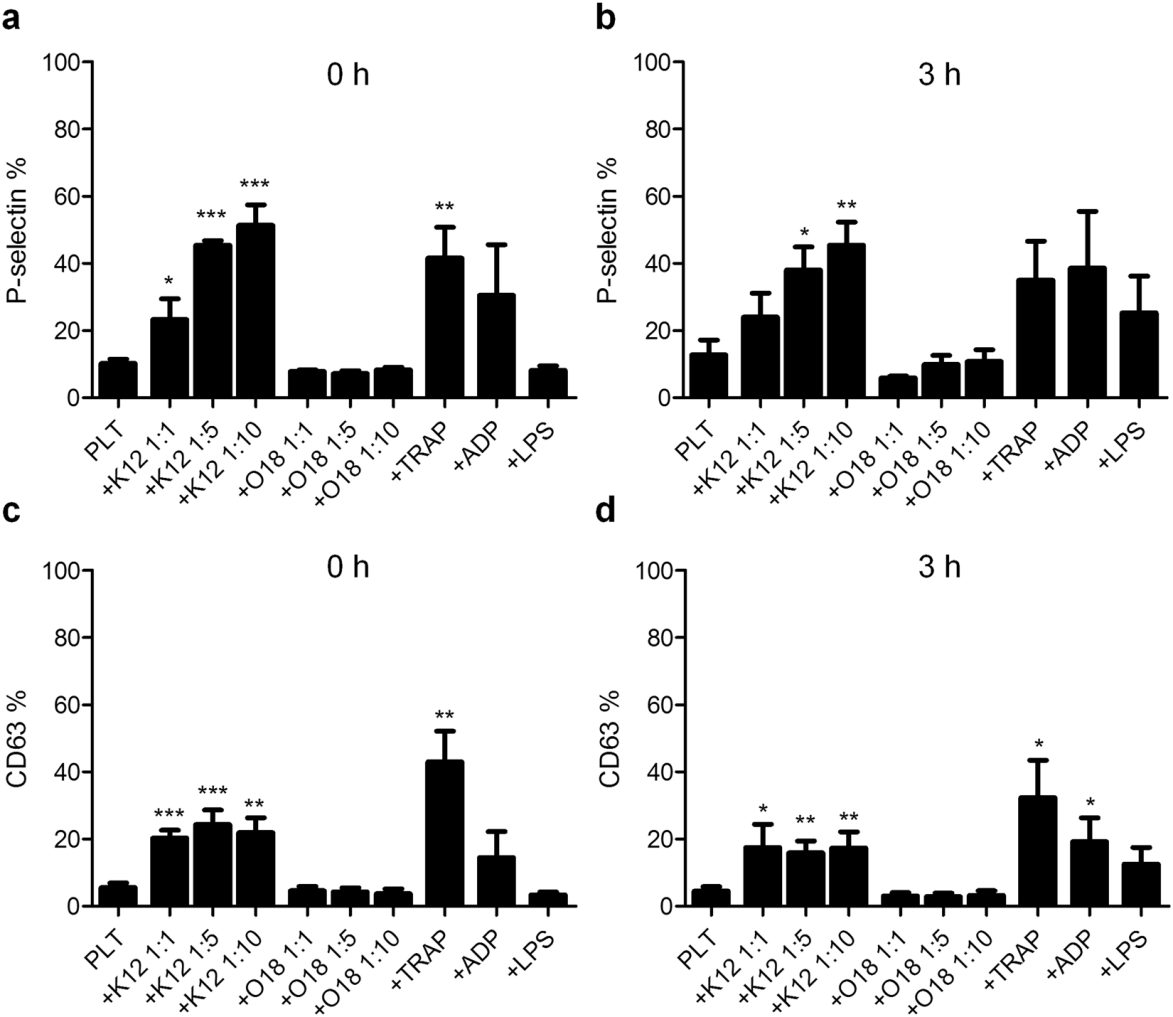
P-selectin and CD63 expression on platelets after co-incubation with bacteria or platelet activators. P-selectin (**a**,**b**) or CD63 (**c**,**d**) were measured on the platelet surface by flow cytometry after gating for the presence of CD41. Platelets incubated without bacteria (PLT) and *E. coli* K12 or O18:K1 co-incubated platelets (platelet-bacteria ratios 1:1, 1:5, 1:10) were analysed at zero hours (**a**,**c**) and after three hours (**b**,**d**) incubation. The activating effect of bacteria was compared to platelet activation by TRAP, ADP or LPS after 15 minutes or three hours incubation time. The data represents percentages (mean ± standard error of the mean) from 3–6 independent experiments. Activation was compared to PLT controls, significance levels are: *p < 0.05, **p < 0.01, and ***p < 0.001. TRAP, thrombin receptor activating peptide 6; ADP, adenosine diphosphate; LPS, lipopolysaccharide.

**Figure 2:**
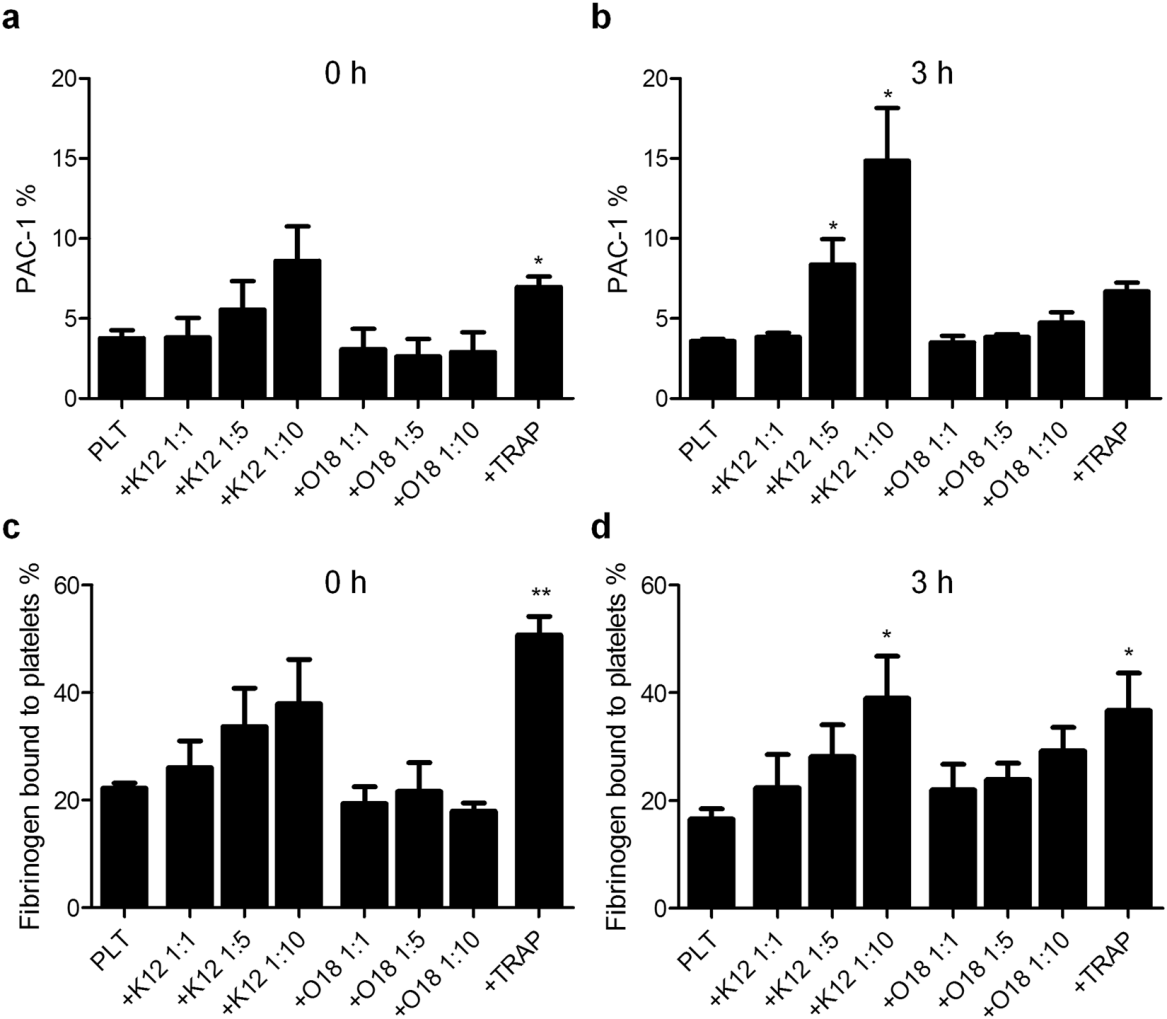
PAC-1 antibody and fibrinogen binding on platelet surface after co-incubation with *E. coli* K12 or O18:K1. PAC-1 antibody (**a**,**b**) and fibrinogen (**c**,**d**) binding were measured on the platelet surface by flow cytometry after gating for the presence of CD61. Platelets incubated without bacteria (PLT) and *E. coli* K12 or O18:K1 co-incubated platelets (platelet-bacteria ratios 1:1, 1:5, 1:10) were analysed at zero hours (**a**,**c**) and after three hours (**b**,**d**). TRAP treated platelets served as a positive control after 15 minutes or three hours incubation time. The data represents percentages (mean ± standard error of the mean) from three independent experiments. Activation was compared to PLT controls, significance levels are: *p < 0.05, **p < 0.01, and ***p < 0.001. TRAP, thrombin receptor activating peptide 6.

The effect of non-pathogenic (K12) and pathogenic (O18:K1) *E. coli* strains on platelet activation was measured by flow cytometry analyses of P-selectin and CD63 expression (Fig. 1), as well as the fibrinogen binding capacity of platelets (Fig. 2).

We observed an increase of P-selectin (24–51.3%, n = 3, p < 0.05) on the platelet’s surface exposed to *E. coli* K12 for either zero or three hours incubation compared to platelets not incubated with bacteria (Fig. 1a,b). P-selectin expression correlated with the number of bacteria added. The effect by *E. coli* K12 was rapid and reached the levels achieved by platelet activators TRAP and ADP already few minutes after addition of the bacteria (zero hours, 1:5 platelet to bacteria ratio).

Similarly, CD63 also increased on the surface of platelets incubated with *E. coli* K12 (15.9–24.3%, n = 3, p < 0.05) compared to platelets not exposed to bacteria. Apparently, this effect was independent of the bacteria concentration (Fig. 1c,d).

Furthermore, we observed that *E. coli* K12 rapidly induced fibrinogen binding to the platelet surface (22.4–39%, n = 3, p < 0.05) compared to control platelets (Fig. 2c,d).

Exposure of platelets to *E. coli* O18:K1 for zero or three hours did not induce a measurable increase of either P-selectin or CD63 on the platelet surface (n = 3) (Fig. 1a–d), and only insignificantly affected fibrinogen (n = 3) (Fig. 2c,d).

The responses of platelets to LPS are still controversial in spite of many attempts for clarification^38,39^. We observed that LPS was only able to induce a minor increase of surface P-selectin after three hours incubation (non-significant).

Also low number of *E. coli* K12 and O18:K1 per platelet (i.e. 10:1 platelet to bacteria ratio) did not increase platelet surface P-selectin or CD63 on co-incubated platelets versus control conditions (data not shown).

### *E. coli* K12 induces activation of the integrin αIIbβ3 receptor

It is essential for platelet aggregation that integrin αIIb and integrin β3 form an active complex (integrin αIIbβIII) to which fibrinogen can bind. The complex formation is detected via binding a PAC-1 antibody, what we evaluated on platelets incubated without or with *E. coli* bacteria.

We observed that platelets exposed to *E. coli* K12 showed PAC-1 positivity already after few minutes (zero hours) of contact between platelets and *E. coli* K12 what corresponded to the number of bacteria added (3.8–8.6%, n = 3, non-significant) (Fig. 2a). The antibody binding was increased after three hours incubation in samples containing a 5-fold or 10-fold bacteria excess over platelets (8.4–14.9%, n = 3, p < 0.05) (Fig. 2b).

*E. coli* O18:K1 did not trigger the binding of PAC-1 antibody (n = 3) (Fig. 2a,b).

Importantly, both bacteria strains were alive, but did not show measurable growth after three hours incubation in SSP + buffer at room temperature (sub-optimal conditions for bacteria growth) compared to zero hours. The presence of washed platelets did not significantly affect the number of *E. coli* K12 or O18:K1 colonies (data not shown).

### *E. coli* K12 differentially alters platelet mRNAs

It has been shown that LPS and a septic environment may affect platelet RNA splicing^8,10^. We measured the influence of external platelet activation by *E. coli* strains on platelet mRNA repertoire by high-throughput mRNA-sequencing.

We incubated platelets isolated from healthy individuals (n = 4) with both *E. coli* strains in 1:1 platelet to bacteria ratio. Platelets of the same healthy individuals incubated without bacteria served as controls. Total RNA was isolated and subjected to RNA-sequencing. We included a poly-A-tailed oligo-dT amplification protocol to minimize the contribution of bacterial RNA to the platelet RNA profiles. We used thromboSeq pipeline^11,12^ to analyse our data. First, an average 9.1 × 10^6^ reads in total per sample (n = 12, (SD) = 1.8 × 10^6^) was observed, from which approximately 6.5 × 10^6^ (n = 12, (SD) = 1.6 × 10^6^) mapped to the human reference genome. We allowed for maximum ten mismatches per 100 base pairs RNA-sequencing reads between our reads and the human reference genome. Of these, 1.2 × 10^6^ corresponded to intron-spanning domains (n = 12, (SD) = 2.6 × 10^5^) (Supplementary Table S1). We specifically investigated intron-spanning RNA reads to uncover the potentially spliced RNA repertoire. We filtered for low abundant RNAs (logCPM >3) and removed those from the dataset. The RNA-sequencing profiles of platelets correlated to previously published platelet RNA-sequencing datasets (Supplementary Fig. S2)^3,6,40^.

**Figure 3:**
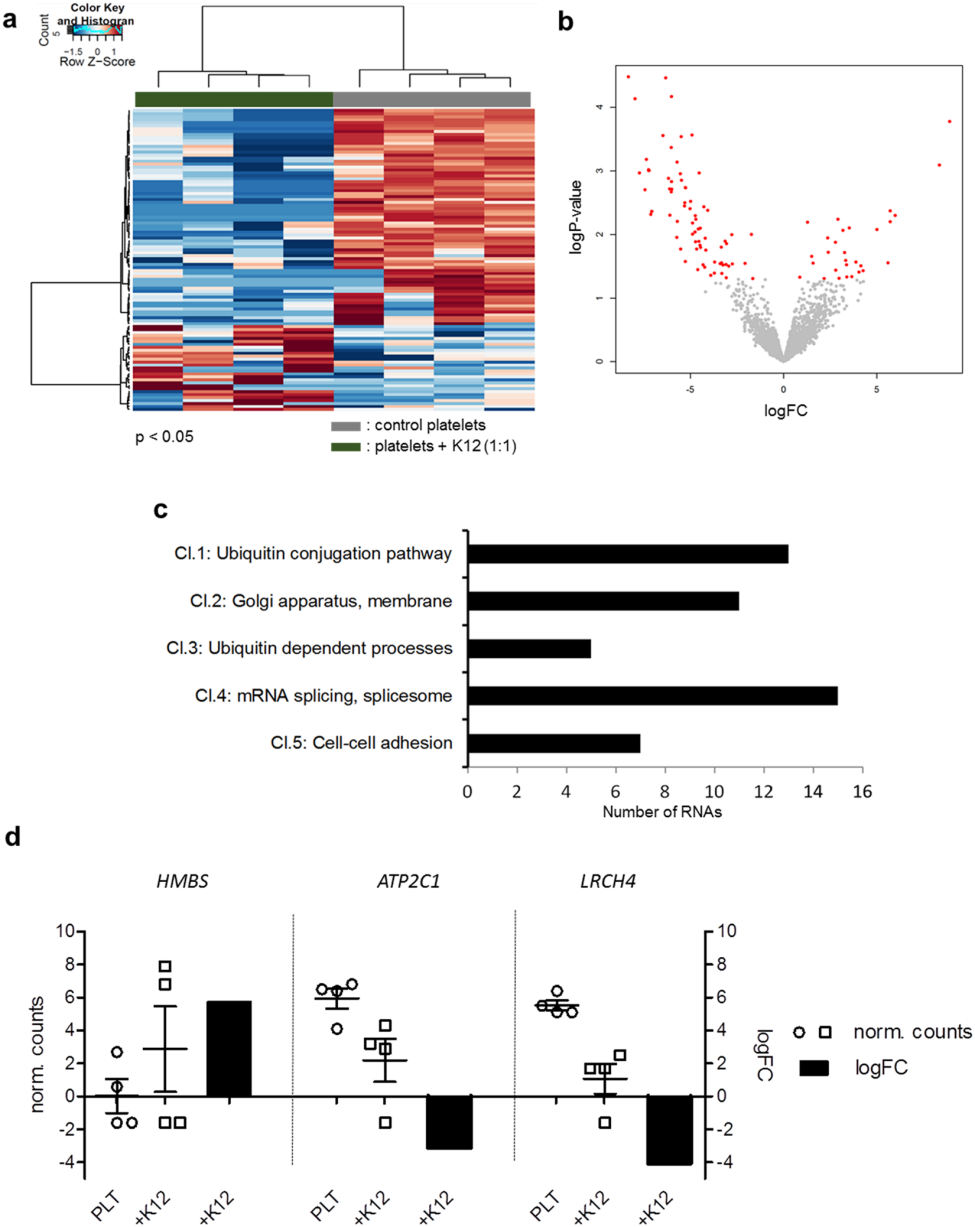
Changes of mRNAs in platelets after exposure to *E. coli* K12. Analysis with thromboSeq pipeline. (**a**) Heatmap of platelet mRNA profiles of control (grey) and *E. coli* K12 co-incubated platelets (green) after three hours incubation in 1:1 platelet-bacteria ratio. (**b**) Volcano plot of mRNAs of control versus *E. coli* K12 (1:1) exposed platelets. RNAs of which the concentrations changed significantly after *E. coli* K12 co-incubation are labelled in red (p < 0.05). (**c**) Gene ontology (GO) analysis of mRNAs with significantly changed concentrations after *E. coli* K12 exposure (p < 0.05) using DAVID functional annotation analysis (cluster enrichment score >1). (d) Logarithm 2 fold change (logFC) and normalized counts (norm. counts) of *HMBS*, *ATP2C1* and *LRCH4* RNAs in platelets exposed to *E. coli* K12 bacteria compared to control platelets. Cl., Cluster.

After *E. coli* K12 exposure 3072 platelet mRNAs were identified with high abundance, whereas 3095 mRNAs were detected after *E. coli* O18:K1 exposure of platelets. Thus, incubation of platelets from four healthy donors with *E. coli* K12 (Fig. 3) or *E. coli* O18:K1 (Supplementary Fig. S3) (1:1 platelet to bacteria ratio, three hours incubation) influenced platelet RNAs.

By ANOVA likelihood-ratio test for detection of differentially expressed RNAs, we could show that exposure of platelets to *E. coli* K12 affected the concentration of 103 spliced RNAs statistically significantly (n = 4 individuals’ platelets incubated without *E. coli*, n = 4 individuals’ platelets incubated with *E. coli* K12, p < 0.05). The level of 29 RNAs was increased (+3.77 average logarithm 2 fold change (logFC)), and of 74 RNAs decreased (−4.98 average logFC) (Fig. 3b). The changes allowed a clear differentiation between pre- and post- *E. coli* K12 contact (p < 0.001 by Fischer’s exact test) (Fig. 3a).

Interestingly, only 21 RNAs which were affected overlapped between *E. coli* K12 and O18:K1 profiles (Supplementary Fig. S3d).

Gene ontology (GO) functional annotation analysis using Database for Annotation, Visualization and Integrated Discovery (DAVID) was performed for all RNAs with significantly changed concentration (p < 0.05) (Fig. 3c, Supplementary Tables S3, S4). We found that incubation of platelets with *E. coli* K12 caused an enrichment of RNAs with the following functional characteristics: involved in splicing (Cluster 4, 15 RNAs), cell-cell adhesion (Cluster 5 with 7 RNAs), related to Golgi apparatus (Cluster 2, 11 members) and ubiquitin related processes (Cluster 1 with 13 and Cluster 3 with 5 RNAs) (cluster enrichment score >1).

When we tested the effects of *E. coli* K12 on platelet RNA concentrations with the Tuxedo RNA-sequencing processing^41–43^ pipeline, we observed significant concentration changes of RNAs, particularly hydroxymethylbilane synthase (*HMBS*), encoding for the protein porphobilinogen deaminase, an enzyme contributing to heme biosynthesis; ATPase secretory pathway Ca^2+^ transporting 1 (*ATP2C1*), a protein involved in calcium ion transport in a magnesium-dependent manner; and leucine rich repeats and calponin homology domain containing 4 (*LRCH4*), translated to a protein with leucine-rich repeats (LRR) involved in ligand binding in both pipelines (Fig. 3d).

We could validate the presence of *HMBS*, *ATP2C1* and *LRCH4* in platelets by RT- PCR analysis. Even though RT-PCR is a semi-quantitative method, we could confirm the same trend of changes of the tested platelet RNAs induced by *E. coli* K12 that we observed by RNA-seqencing. Using human-sequence specific RT-PCR primers, we made sure that the changes are independent of potential *HMBS*, *ATP2C1*, and *LRCH4* production by bacteria (data not shown).

*HMBS* RNA^44^ as well as *ATP2C1* RNA and protein^45,46^ have been reported to be present in platelets, but their functions remain largely unclear. The presence of *LRCH4* had not been described in platelets yet. Until now, the effect of *E. coli* on these RNAs in platelets has not been investigated. Our data indicates that exposure of platelets to *E. coli* K12 results in an increase of *HMBS* (logFC = +5.73), and a decrease of *ATP2C1* (logFC = −3.13) and *LRCH4* (logFC = −4.07) RNA.

### Effect of *E. coli* on HMBS, ATP2C1 and LRCH4 proteins in platelets

Alterations of platelet mRNA may influence the platelet protein content as well.

**Figure 4:**
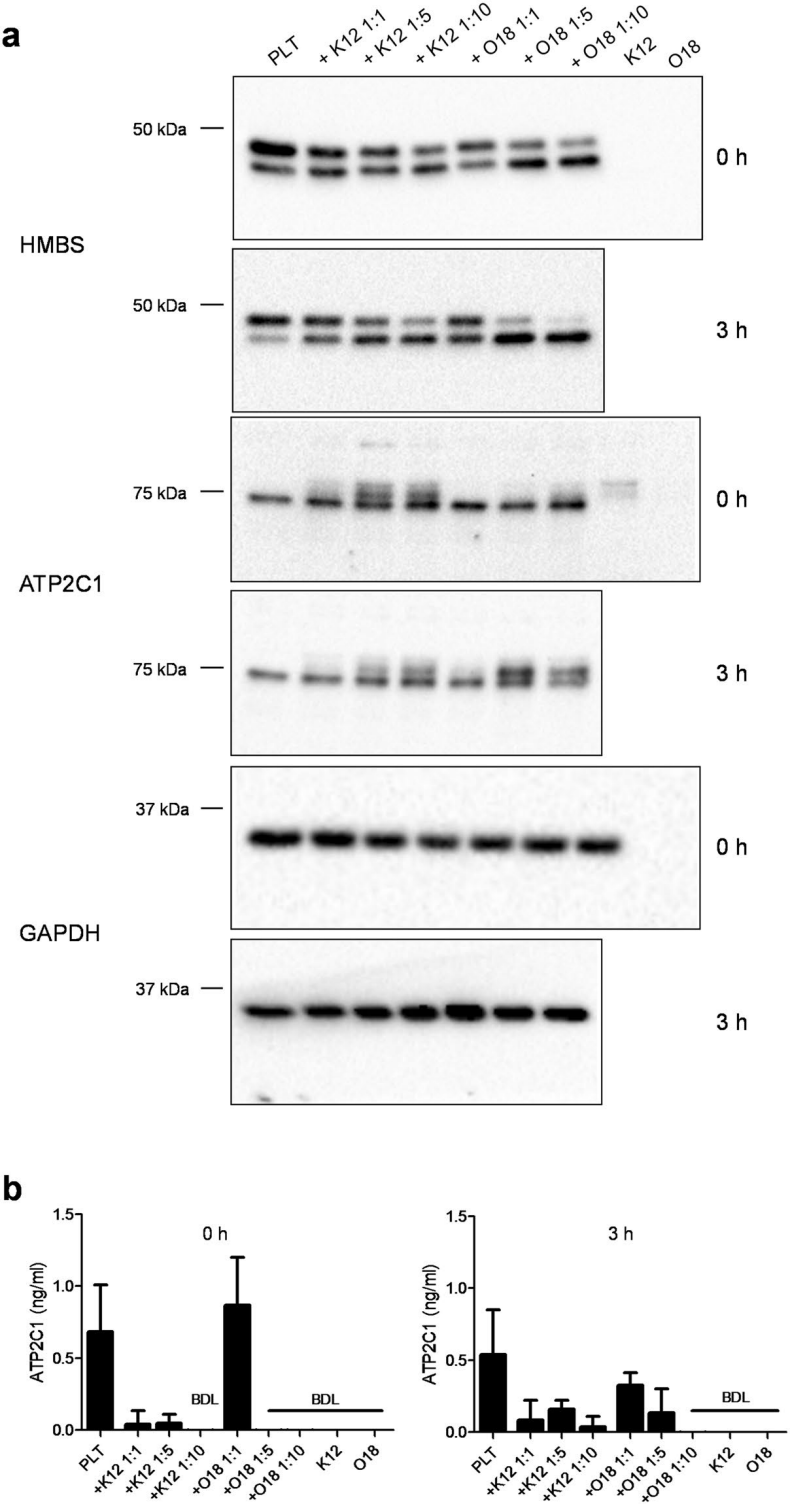
Western blot and ELISA analysis of HMBS and ATP2C1. (**a**) Cell lysates were applied to SDS-PAGE gels under reducing conditions. HMBS and ATP2C1 protein were detected in platelets using a polyclonal antibody. Incubation of platelets with *E. coli* K12 and *E. coli* O18:K1 in 1:5 or 1:10 platelet-bacteria ratios converted HMBS 47 kDa form to a 40 kDa protein. ATP2C1 was not affected. GAPDH was used as a loading control. (**b**) The releasates of the platelet-bacteria mix were collected after centrifugation (500 g, 10 minutes without break). HMBS, ATP2C1 and LRCH4 levels were measured by ELISA. Data represents the mean of three independent experiments (n = 3). ATP2C1 was detectable in platelet supernatants, while HMBS and LRCH4 proteins were either not released from platelets or in concentrations below the detection level of the ELISA (data not shown). BDL, below detection limit. Western blot results are representative image of three replications. The same exposure was applied equally across the entire image. The original pictures of the full-length western blots can be found in Supplementary Fig. S7a, b, c, d, g, h.

By Western blot analysis we could demonstrate the presence of HMBS, ATP2C1 and LRCH4 proteins in platelets before and after *E. coli* K12 exposure (in 1:1, 1:5 and 1:10 platelet to bacteria ratios) (Fig. 4a, Supplementary Fig. S4).

We detected no visible concentration difference or molecular weight change of ATP2C1 and LRCH4 in platelets after *E. coli* K12 exposure. In contrast, when present in 5-fold or 10-fold excess over platelets, *E. coli* K12 converted HMBS from a 47 kDa form to a 40 kDa molecular weight protein.

**Figure 5:**
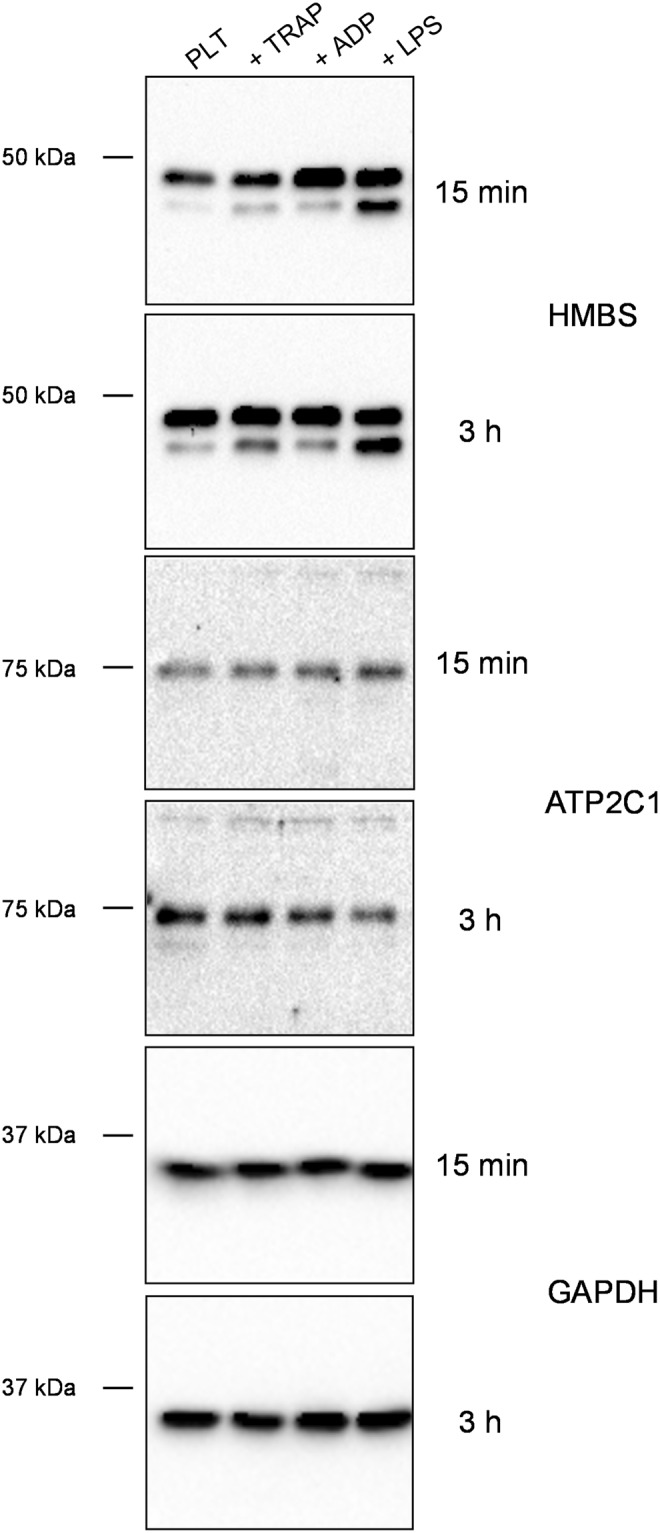
Effect of platelet activators on HMBS and ATP2C1 proteins. Electrophoretic migration behaviour of HMBS and ATP2C1 was analysed in platelets treated with different activators. The molecular weight of HMBS in platelets was changed by LPS similar to the changes caused by *E. coli* K12 and O18:K1. TRAP and ADP had no effect on the molecular weights of HMBS and ATP2C1. TRAP, thrombin receptor activating peptide 6; ADP, adenosine diphosphate; LPS, lipopolysaccharide. Western blots show representative images of three replicative experiments. The same exposure was applied equally across the entire image. The original pictures of the full-length western blots can be found in Supplementary Fig. S7i, j, k, l, m, n, o, p.

We also evaluated the effect of *E. coli* O18:K1 (Fig. 4a, Supplementary Fig. S4) as well as of the platelet agonists TRAP, ADP and LPS on the HMBS, LRCH4 and ATP2C1 proteins (Fig. 5, Supplementary Fig. S5). We saw that *E. coli* O18:K1 and LPS converted HMBS to the 40 kDa form. Interestingly, LPS had little influence on platelet activation of washed platelets (Fig. 1). Neither *E. coli* O18:K1, nor the three platelet activators had any effect on the protein levels or molecular weight of ATP2C1 or LRCH4.

### ATP2C1 seems to be released from platelets; *E. coli* exposure seems to reduce the release

It is known, that platelets can sequester^1,2^ and release^47,48^ RNA and proteins. By ELISA analysis of the supernatant obtained after incubation of platelets in buffer for zero and three hours, we observed ~0.5 nanogram/milliliter of ATP2C1 protein in the supernatant (Fig. 4b). This potential release of ATP2C1 seems to be reduced by the interaction of platelets with *E. coli* K12 and O18:K1, especially when measured at three hours post-incubation. HMBS or LRCH4 could not be detected in the supernatant of platelets or platelets exposed to bacteria with the applied ELISA assays (data not shown).

## Discussion

Platelets are able to interact with different bacteria directly via platelet surface receptors such as P-selectin, glycoproteins^49^ and TLR4^50^, indirectly through plasma proteins^51^, or via bacteria released exotoxins^52^. The interaction with *E. coli* can be mediated through FcγRIIa, integrin αIIbβ3^36^ and shiga toxin^53^ or LPS via TLR4^54^, however the latter one seems to be dependent on the surface structure of the bacteria.

We observed that platelet exposure to *E. coli* K12 led to an increased surface expression of the activation markers P-selectin and CD63. Presumably, platelets release contents of α- granules and dense bodies upon interaction with this bacteria strain. Furthermore, *E. coli* K12 also triggers PAC-1 antibody and fibrinogen binding to integrin αIIbβ3 on platelets in a bacteria concentration dependent manner. The presence of active integrin αIIbβ3 on the platelet surface is a prerequisite for platelet aggregation. Interestingly, the pathogenic *E. coli* O18:K1 was not able to induce an increase of the platelet surface activation markers and it also did not significantly affect integrin αIIbβ3 activity.

A possible explanation for the difference between the pathogenic and non-pathogenic *E. coli* may be found in the surface of the two strains. *E. coli* strains carrying an O side-chain on the surface besides the lipid A and core oligosaccharides^55^ are denoted as “smooth”, like the pathogenic *E. coli* O18:K1. In contrast, “rough” strains lack the O-antigen, such as the non-pathogenic *E. coli* K12. *In vitro* studies show differences in the activity of the two types of LPS, with “rough” LPS activating a broader range of cells with higher efficiency compared to the “smooth” form. It has been proven, that macrophages are able to respond to “rough” LPS and lipid A, but not to “smooth” LPS^56^. The “smooth” form requires CD14 to activate immune cells^57^. Possibly, the same holds true for platelets *in vitro*. Studying the contribution of plasma proteins to platelet - *E. coli* interaction would be an interesting future aspect to consider.

In *in vivo* comparisons, several studies showed an involvement of the complement system in LPS - induced platelet granular release and accumulation. It was found that O-antigen is important, but is not the only virulence factor influencing platelet activation^57–59^. The fact that in spite of its ability to activate platelets *E. coli* K12 is non-pathogenic while O18:K1 cannot activate washed platelets but is pathogenic may be associated with the sensitivity to the complement system. While *E. coli* K12 is a complement sensitive strain, *E. coli* O18:K1 is complement resistant and uropathogenic. The O antigen on the surface reduces activation of the alternative complement pathway, and the K1 antigen of this strain inhibits the classical complement pathway^60,61^. The complement resistance may contribute to the survival of the bacteria strain in the blood stream.

We showed that both *E. coli* strains were able to induce alterations in the platelet RNA repertoire. The *E. coli* K12 exposure lead to a decreased expression of 74 out of 103 RNAs in comparison to platelets not co-incubated with *E. coli* K12.

DAVID GO functional annotation analyses revealed an enrichment of groups of RNAs with common functional characteristics in platelets after incubation with *E. coli* K12 (cluster enrichment score >1) (Fig. 3c). In Cluster 2, Golgi apparatus and membrane related RNAs were enriched. It is known, that in platelets Golgi elements support selective modification and secretion of molecules, like enzymes or sugar nucleotides^62^. It has been shown that blocking of the Golgi apparatus may result in decreased CD40L expression^63^. *ATP2C1*, of which the RNA concentration is decreased by the presence of *E. coli*, is found in this cluster. *ATP2C1* has a role in Ca^2+^/Mn^2+^ transport and membrane trafficking, localized on the Golgi apparatus. It has been reported that a deficiency of the protein induces defects in secretory pathways^64^. Interestingly, RNAs annotated to ubiquitination processes, GPVI activation and platelet survival were also affected^65–67^. Platelets with an impaired secretion system cannot be activated and cannot release microbicidal components or activate other immune cells. This may result in improved survival of bacteria in the blood stream. Cluster 4 comprises 15 RNAs involved in mRNA splicing. Perhaps, platelet - *E. coli* K12 interaction influences splicing and translation of proteins affecting RNA splicing, however that needs further investigations. Interestingly, RNAs annotated to cell-cell adhesion (Cluster 5) were also enriched. This suggests that cell adhesion mechanisms occurring prior to platelet activation^19^ may be induced by *E. coli*. A number of other possibly relevant RNAs were also affected.

The analysis of platelet RNA after exposing platelets to bacteria for three hours provides an interesting insight into RNA concentration changes. However, including further time points (e.g. 15 minutes, one hour) will reveal more information about the expression kinetics of different RNA subsets. Besides, inter-individual variation might result in differential platelet reactivity. Kinetic analysis might also add knowledge on the differences experienced between individuals (Fig. 3d).

It is known that changes in RNA concentrations are not always paralleled by a change of expression of the corresponding proteins^68^. Possibly, a reduced level of a spliced RNA indicates either translation to protein, or release of this RNA into the supernatant, or degradation of the RNA. Therefore, we evaluated whether HMBS, ATP2C1 and LRCH4 proteins were present and/or affected in platelet lysates and supernatants after exposure to *E. coli* strains.

In humans, two isoforms of HMBS enzyme have been described: a ubiquitously expressed “housekeeping” form^69^ and a shorter erythroid form which is apparently only present in erythropoetic cells^70^. The role of HMBS in platelets remains so far unclear. The two isoforms are transcribed from a single gene, but encoded by two different, alternatively spliced mRNAs. Surprisingly, we did not observe the two alternatively spliced mRNAs, but identified two protein forms in platelets. Incubation of platelets with *E. coli* or with LPS very quickly converts the larger into the shorter form. We can only speculate that HMBS may be relevant for platelet-bacteria interaction via the LPS pathway.

Interestingly, we found that ATP2C1 was released from platelets incubated without bacteria (~0.5 ng/ml). This release happened almost immediately after the start of the incubation, but decreased in platelets co-incubated with *E. coli* K12 and O18:K1. The decrease was more pronounced in the presence of a higher amount of both bacteria strains (1:5 and 1:10 platelet to bacteria ratio). The effect of *E. coli* on the release of ATP2C1 could represent a defence mechanism, and it cannot be excluded that similar effects may exist for other platelet-derived proteins. The reduced levels of *ATP2C1* RNA in the platelets (Fig. 3d) together with the reduced protein levels in the supernatant could also indicate an active translation of ATP2C1 protein and retention in the platelets in the presence of bacteria, even though the qualitative Western blot analysis did not show major differences of this protein in platelets incubated without or with *E. coli*. To clarify what happens to *ATP2C1* RNA, protein and its secretion in the presence of bacteria will require further studies.

Our results show that the changes of platelet RNA levels induced by bacteria cannot be directly translated to the pathogenicity status of bacteria. Non-pathogenic strains which do not trigger sepsis or bacteraemia, can still cause changes in platelet RNA and protein profiles, as we could show for *E. coli* K12; and even though the pathogenic *E. coli* O18:K1 was not able to induce an increase of platelet surface activation markers, it significantly affected platelet RNAs and certain proteins. We suspect that the altered RNA and protein expressions could affect platelets or other immune cells. Further investigations regarding platelet RNA and protein analysis will be necessary for a more detailed understanding of platelet function in bacterial infections, for instance urinary tract infection or sepsis.

## Methods

The study was approved by the Ethics Committee of the Medical University of Vienna and all research was performed according to the ethical guidelines.

### Isolation of platelets

Citrated blood of healthy donors was used to isolate platelets. The blood was collected at the Department of Transfusion Medicine (Medical University of Vienna), and all donors gave written informed consent. Platelet rich plasma (PRP) was prepared using centrifugation at 150 g for 15 minutes at room temperature. Since immune-depletion of platelet suspension is not suitable for RNA profiling, PRP was applied to an Optiprep (Axis-Shield, Oslo, Norway) density gradient and spun at 350 g for 15 minutes to decrease the leukocyte number of the suspension. After harvesting the platelet layer, cells were washed with HEPES-Tyrode buffer (10 mM HEPES, 137 mM NaCl, 2.8 mM KCl, 1 mM MgCl2,12 mM NaHCO3, 0.4 mM Na2HPO4, 5.5 mM glucose, and 0.35% bovine serum albumin [BSA]). Centrifugation was performed without break, and before every centrifugation step, 400 nM prostaglandin I_2_ (PGI_2_) (Sigma-Aldrich, Munich, Germany) was added to prevent platelet activation. Washed platelets were resuspended in SSP + buffer (Macopharma SA, Turcoing, France) and kept on a see-saw shaker (10 rpm) for one hour before starting the experiments. Platelet and leukocyte counts were determined on a Sysmex XE-2100 (Sysmex, Kobe, Japan) instrument.

After optimizing the isolation protocol, the leukocyte contamination (CD45 positivity) of platelet suspensions was tested with qPCR. Leukocyte contamination was determined using a standard curve prepared with platelets spiked with a different number of leukocytes. Occasional tests of the platelet suspensions showed less than 1 leukocyte/10^5^ platelets, which was considered adequate for RNA sequencing. All samples contained less than 15% P-selectin positive platelets after isolation.

### Incubation of washed platelets with bacteria

Two different strains of *E. coli* bacteria were used: the non-pathogenic *E. coli* K12 C600 (purchased from the Coli Genetic Stock Centre; Yale, CT, USA) and the uropathogenic *E. coli* O18:K1 (patient isolate, gift from S. Knapp; Medical University of Vienna). The bacteria were grown in lysogeny broth (LB) (Sigma-Aldrich) at 37 °C and 160 rpm until they reached an OD600 nm of 0.5–0.9. To determine the number of bacteria in the suspensions, OD measurements were performed in suspensions in which the number of colony forming units (CFU)/ml was known. With these data, standard curves were prepared. The bacteria cells were pelleted by centrifugation at 18 000 × g for 10 minutes and washed with SSP + buffer. After washing, the platelet pellet was resuspended in SSP + and used for *in vitro* exposure to the bacteria in 1:1, 1:5 or 1:10 platelet-bacteria ratio. Incubation was done on a see-saw shaker (10 rpm) at room temperature.

In order to monitor the bacteria numbers and physiological status during incubation, platelets, platelets co-incubated with *E. coli* K12 or O18:K1 (in 1:1, 1:5 and 1:10 platelet to bacteria ratio) and bacteria alone as controls (same number of bacteria as mixed with platelets) were plated on LB agar plates after appropriate dilution at zero hours and three hours. The agar plates were incubated overnight on 37 °C and the number of colonies was counted. The CFU/ml were calculated and the different time points and conditions were compared to each other.

### Platelet analysis by flow cytometry

Platelets were identified by expression of CD41 (APC, Clone: HIP8) or CD61 (FITC, Clone VI-PL2). To determine possible platelet activation following incubation with bacteria, P-selectin (from alpha granules) and CD63 (from dense bodies) expression on the platelet surface was measured by flow cytometry. To test integrin αIIbβ3 activation on the platelet surface PAC-1 antibody and antibody-labelled fibrinogen were used. At baseline (zero hours) and after three hours incubation the platelet-bacteria mixtures (5 × 10^6^ platelets in 1:1, 1:5 or 1:10 platelet to bacteria ratios) were fixed with 0.5% formalin (Roth, Karlsruhe, Germany), mixed with antibodies against CD41 (APC, Clone: HIP8), P-selectin (PE, Clone: AK4) and CD63 (FITC, Clone: H5C6) (BioLegend, San Diego, CA, USA) for 30 minutes and again fixed with 1% formalin. For the PAC-1 (FITC, Clone: PAC-1) (BioLegend, San Diego, CA, USA) and fibrinogen (from human plasma, Alexa Fluor 647 Conjugate) (Thermo Fisher Scientific, Waltham, MA, USA) assays, the staining procedure was performed without the first fixation step gating for CD61 (FITC, Clone VI-PL2) (BioLegend, San Diego, CA, USA) positive platelets during the measurement. All samples were measured after staining using a BD FACSCalibur flow cytometer. Analysis was performed with the FlowJo (TreeStar, Ashland, OR, USA) software, and visualized by Graph Pad Prism version 5.01. for Windows (GraphPad Software, La Jolla, CA, USA. www.graphpad.com).

The following platelet activators were used as positive controls: thrombin receptor-activating peptide 6 (TRAP, 14.5 µM; Roche, Penzberg, Germany), adenosine diphosphate (ADP, 5 µM; Roche) and lipopolysaccharide O111:B4 (LPS, 3 µg/ml; Sigma-Aldrich).

### RNA isolation

Incubation of washed platelets with bacteria (5 × 10^8^–10^9^ platelets in 1:1 platelet to bacteria ratio, for three hours) was followed by centrifugation of the samples at 500 g for 10 minutes. The pellets were used for RNA isolation with a standard phenol-chloroform procedure. Briefly, centrifuged cells were resuspended in 500 µl Trizol reagent (Invitrogen, Life Technologies, Carlsbad, CA, USA), and added to Phase lock tubes (QuantaBio, Beverly, MA, USA) together with chloroform. All centrifugation steps were performed as previously described^26^. The RNA pellet was air-dried and resuspended in 40 µl RNAse free water.

### RNA-sequencing library preparation and sequencing

Preparation of RNA samples for sequencing was performed as described by Best *at al*.^12^. RNA quality and quantity was tested using the RNA 6000 Picochip (Bioanalyzer 2100, Agilent, Santa Clara, CA, USA) (Supplementary Fig. S1). For cDNA synthesis and amplification ~600 pg total platelet RNA was added to the SMARTer Ultra Low RNA Kit for Illumina Sequencing v3 (Clontech, Mountain View, CA, USA). The bacteria co-incubated platelet samples contained higher RNA concentrations, which reflected bacterial RNA contribution to the total RNA. To make sure that we applied the same amount of platelet RNA from samples incubated without or with bacteria, we corrected for the bacteria RNA content in our co-incubated samples and added ~1200 pg total RNA to the SMARTer preparation from the bacteria exposed samples. The chosen amount of total RNA was based on exemplary sequencing results. Following a cDNA quality test on a DNA High Sensitivity chip (Agilent), cDNA samples were sonicated (Covaris Inc., Woburn, MA, USA) for nucleic acid shearing. DNA was labelled with the Truseq Nano DNA Sample Prep Kit (Illumina, San Diego, CA, USA). The quality check was performed on DNA 7500 chips (Agilent). Then, a 100 bp Single-Read sequencing on the Illumina Hiseq. 2500 platform was carried out.

### Quantification and statistical analysis of RNA-sequencing data with thromboSeq pipeline

The FASTQ-files of the raw RNA-sequencing data were processed using a standardized, platelet optimized RNA-sequencing alignment pipeline as previously described^11,12^. Trimmomatic (version 0.22) was used to clip the sequencing adapters and perform 5′-end quality trimming^71^. Mapping to the human reference genome (hg19) was performed with STAR (version 2.3.0, number of mismatches allowed per read: 10)^72^, and HTseq (version 0.6.1) was applied to summarize the intron-spanning reads. R (version 3.3.0) and R-studio (version 0.99.902) were used to perform normalization, statistical and analytical analyses, particularly using the edgeR package^73^. Gene ontology (GO) analysis was done with Database for Annotation, Visualization and Integrated Discovery (DAVID) 6.8 functional cluster annotation^74^. Platelet specific functional analyses of RNA-sequencing data was performed with PlateletWeb database^75^.

### RNA-sequencing analysis with Tuxedo pipeline

RNA-sequencing analysis was also performed with the Tuxedo pipeline. For each sample, RNA-sequencing reads passing vendor quality filtering was aligned to the hg38 reference genome assembly with the TopHat2 (version 2.1.1)^41^. Cufflinks (version 2.1.1)^43^ was used for transcriptome assembly, customary including novel transcript structures, on the basis of the reference transcriptome and spliced read alignments, as well as raw transcript quantification. Differential expression was performed with Cuffdiff (included in Cufflinks version 2.1.1)^42^ and R scripts were used to perform quality assessment and further refine analysis results. The results were compared to the data produced by thromboSeq pipeline.

### Western blot protein analysis

Following incubation (without or with bacteria in 1:1, 1:5 or 1:10 platelet to bacteria ratios or activators) platelets were pelleted via centrifugation (at 500 g for 10 minutes, without break) and lysed with RIPA buffer containing 2% SDS (Sigma-Aldrich) and 100 µl Halt^TM^ Protease Inhibitor Single-Use Cocktail (Thermo Scientific, Boston, MA, USA). The total protein concentration was determined by bicinchonic acid protein assay (Thermo Scientific) and we controlled how significantly bacteria cells would influence it. We did not observe significant contributions of the bacteria to the measured total protein concentration in samples incubated without or with bacteria. Therefore we were confident that we could use an aliquot of 18 µg protein for electrophoresis on 8% polyacrylamide gel SDS-PAGE for all platelet samples. Gels were blotted as described before^26^. The blots were incubated overnight with primary anti-human antibodies (rabbit polyclonal ATP2C1 (1:750), LRCH4 (1:500), HMBS (1:1000) or GAPDH (1:600) from Atlas Antibodies, Bromma, Sweden). Goat anti-rabbit (H + L) horseradish peroxidase (HRP) conjugate (1:50000; BioRad, Hercules, CA, USA) served as secondary antibody. Signal development was performed by ECL West Pico and West Femto detection system (Thermo Scientific); imaging was done with a Bio-Rad Imaging System. The molecular weights of HMBS, ATP2C1 and LRCH4 proteins in platelets were compared to the molecular weights of the proteins in HeLa cells (Supplementary Fig. S6).

### Enzyme-linked immunosorbent assay (ELISA)

Platelet releasates were collected from platelets incubated with buffer or bacteria (10^8^ – 5 × 10^8^ platelets in 1:1, 1:5 or 1:10 platelet to bacteria ratios) and used for ELISA analyses. Commercially available kits detecting HMBS, ATP2C1 and LRCH4 (DlDevelop, Wuxi, China) proteins were applied. Aliquots of 100 µl undiluted supernatants were analysed. The protocols provided by the manufacturers were followed.

The datasets generated during the current study are available in the Gene Expression Omibus (GEO) database repository (https://www.ncbi.nlm.nih.gov/geo/query/acc.cgi?acc=GSE114710).

## Electronic supplementary material

Supplementary material
