# Supplementary material for: Impact of Escherichia coli K12 and O18:K1 on human platelets: Differential effects on platelet activation, RNAs and proteins
Source: Sci Rep. 2018 Nov 1;8:16145. doi: 10.1038/s41598-018-34473-w (PMC6212526; doi:10.1038/s41598-018-34473-w)
Supplement: Supplementary file 1 — Supplementary material [file 41598_2018_34473_MOESM1_ESM.pdf]

Supplementary information

Impact of *Escherichia coli* K12 and O18:K1 on human platelets. Differential effects on platelet activation, RNAs and proteins.

A. V. Fejes[1], M. G. Best[2,3,4], W. A. van der Heijden[5], A. Vancura[2,4], H. Verschueren[2,4], Q. de Mast[5], T. Wurdinger[2,4], and C. Mannhalter[1] *

[1]Department of Laboratory Medicine, Medical University, Vienna, 1090, Austria
[2]Department of Neurosurgery, Cancer Center Amsterdam, Amsterdam UMC, Vrije Universiteit Amsterdam, Amsterdam, 1081 HV, the Netherlands
[3]Department of Pathology, Cancer Center Amsterdam, Amsterdam UMC, Vrije Universiteit Amsterdam, Amsterdam, 1081 HV, the Netherlands
[4]Brain Tumour Center Amsterdam, Amsterdam UMC, Vrije Universiteit Amsterdam, Amsterdam, 1081 HV, the Netherlands
[5]Department of Internal Medicine, Radboud University Medical Center, Nijmegen, 6500 HB, the Netherlands
*christine.mannhalter@meduniwien.ac.at

Supplementary figures

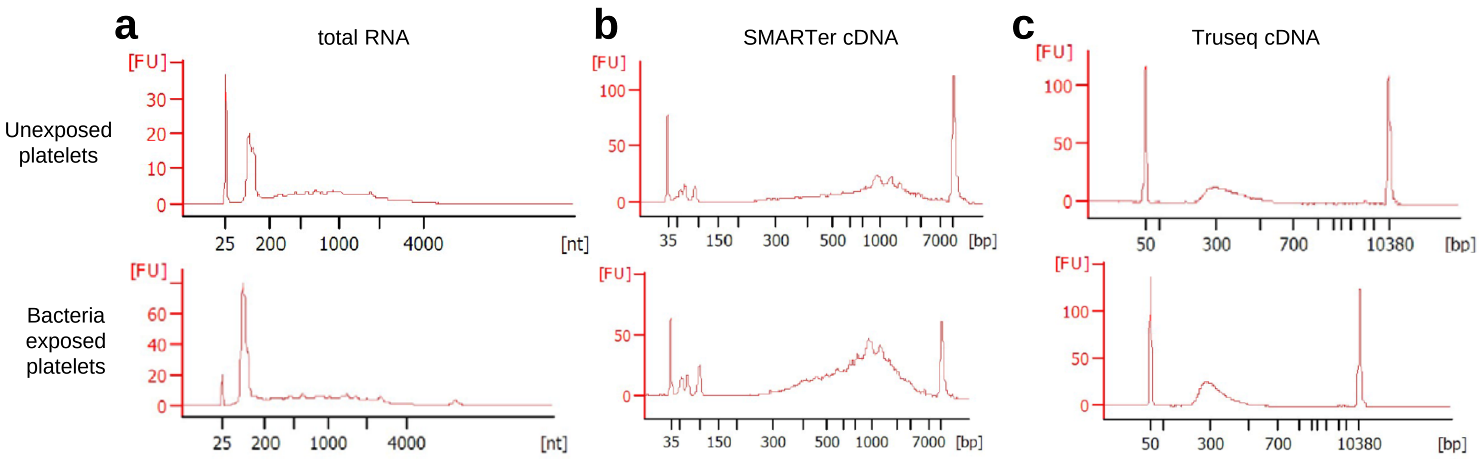

Supplementary Figure S1. Bioanalyzer profiles of control and bacteria treated samples for total RNA measured by RNA 6000 Picochip (a), SMARTer amplified cDNA measured by DNA High Sensitivity chip (b) and Truseq cDNA library measured by DNA 7500 chip (c).

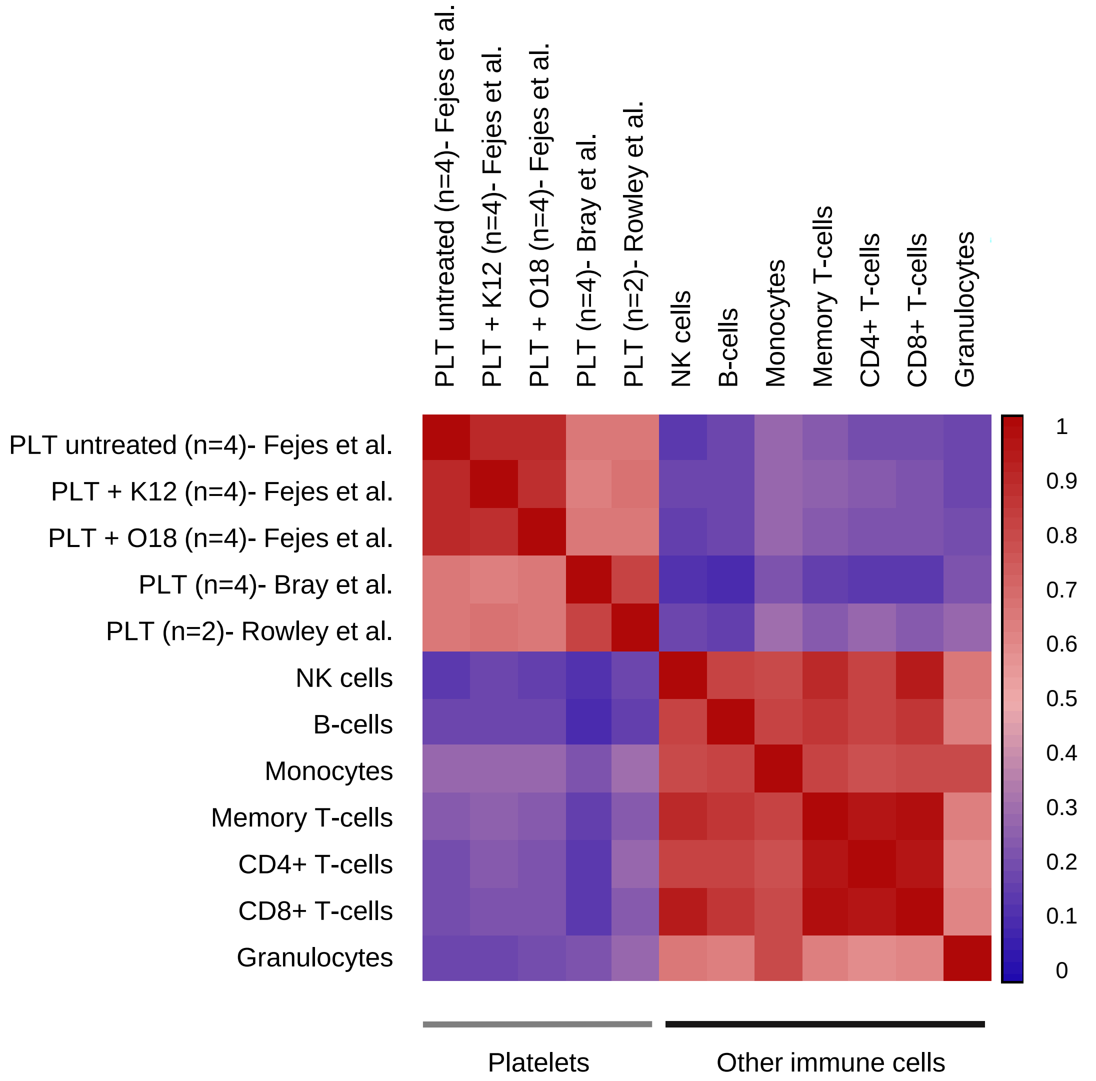

Supplementary Figure S2. Correlation matrix (Pearson correlation, color bar) of sequenced platelet samples (row 1-3, Fejes et al.) and publicly available datasets of platelets (row 4-5, Bray et al. and Rowley et al.) and other immune cells (row 6-12, Hrdlickova et al.). All platelet samples correlated while we did not observe correlation with other immune cells. The differences between our platelet samples and the publised platelet datasets may originate e.g from the different platelet isolation protocols and incubation times. The number of individuals used in the datasets was noted, if applicable. PLT, platelet.

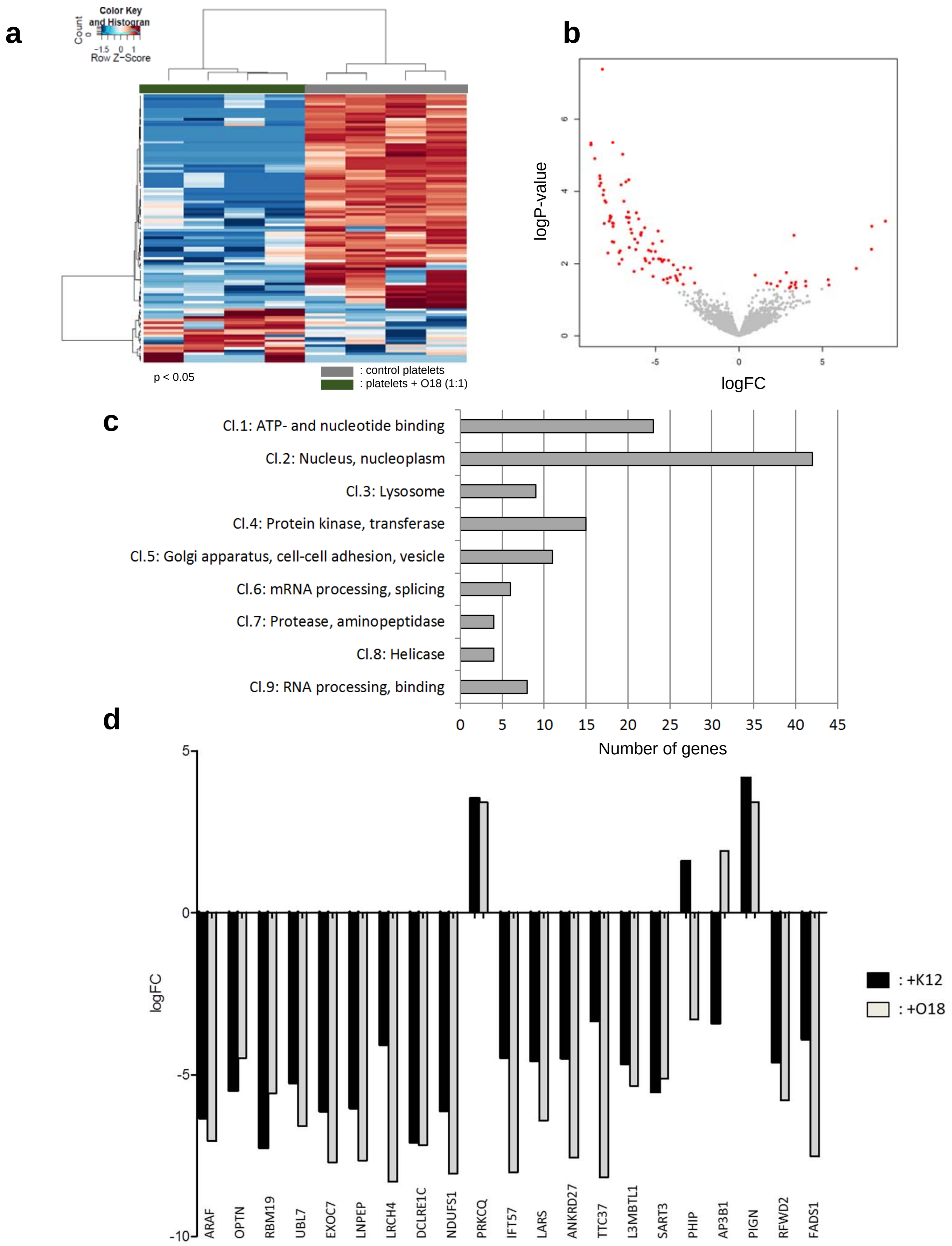

Supplementary Figure S3. (a) Heatmap of platelet RNA profiles of control (grey) and *E. coli* O18:K1 exposed platelets (green) at 3h incubation time in 1:1 platelet to bacteria ratio. (b) Volcano plot of spliced RNA logarithmic fold changes upon *E. coli* O18:K1 (1:1) exposure in platelets. RNAs labelled in red are significantly changed after bacteria co-incubation ($p < 0.05$). (c) Gene onthology (GO) analysis of significantly changed spliced RNAs of platelets followed by *E. coli* O18:K1 exposure ($p < 0.05$) using DAVID functional annotation analysis (cluster enrichment score > 1). (d) Overlapping, significantly changed RNAs between *E. coli* K12 and O18:K1 exposed bacteria platelet profiles. This graph shows the 21 platelet RNAs that were affected by exposure to *E. coli* K12 as well as O18:K1 bacteria. Detailes are shown in Supplementary Table S2. Cl., cluster; logFC, logarithmic 2 fold change.

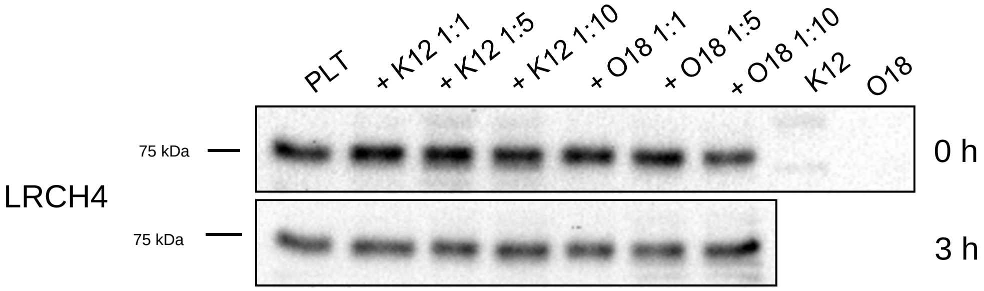

Supplementary Figure S4. Evaluation of LRCH4 protein by Western blot. Cell lysates were applied on SDS-PAGE gel under reducing conditions. LRCH4 protein was detected in platelets, but bacteria did not affect the protein concentration. GAPDH was used as a loading control. The original pictures of the full-length western blots can be found in Supplementary Fig. S7e, f.

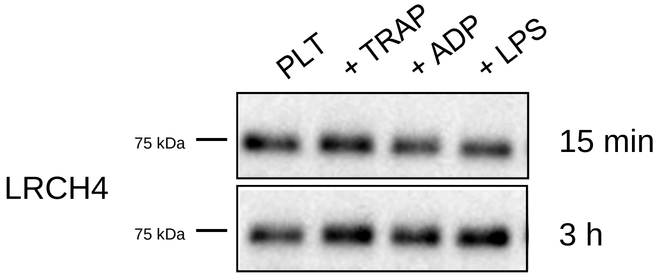

Supplementary Figure S5. Effect of platelet activators on LRCH4 protein. By Western blot, LRCH4 protein was analyzed in platelets treated with different activators. The activators did not change LRCH4 protein concentration nor they did the migration behaviour. TRAP, thrombin receptor activating peptide 6; ADP, adenosine diphosphate; LPS, lipopolysaccharide. The original pictures of the full-length western blots can be found in Supplementary Fig. S7m, n.

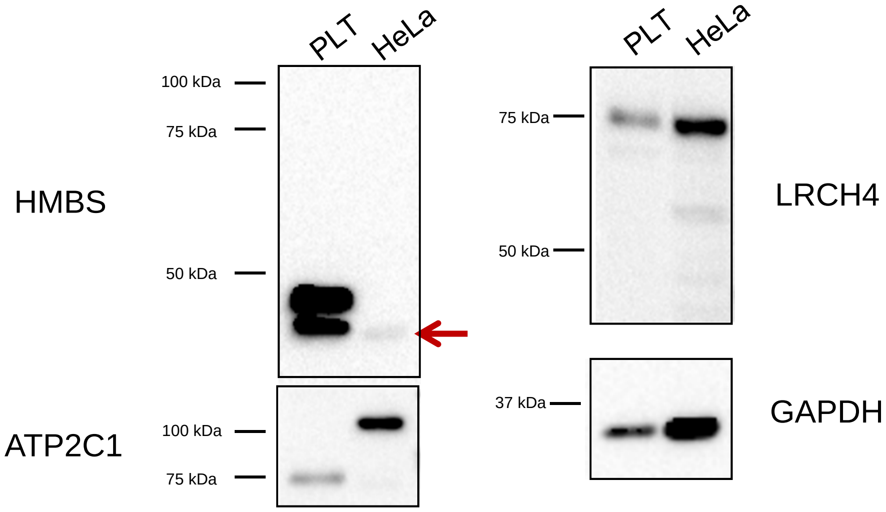

Supplementary Figure S6. HMBS, ATP2C1 and GAPDH in control platelets (PLT) and HeLa cells. An aliquot of 18 ug protein isolated from platelets or HeLa cells was applied to 8% polyacrylamide gels and analyzed by Western blots. With HMBS antibodies we detected only a very weak band at 40 kD in HeLa cells while we saw two intensive bands at 47 kDa and 40 kDa in platelets. The LRCH4 protein showed the same mobility in platelets and HeLa cells. ATP2C1 migration was different in platelets (75 kDa) and HeLa cells (105 kDa). The original pictures of the full-length western blots can be found in Supplementary Fig. S7q, r, s, t.

Supplementary Figure S7. Unaltered, full-length Western blots

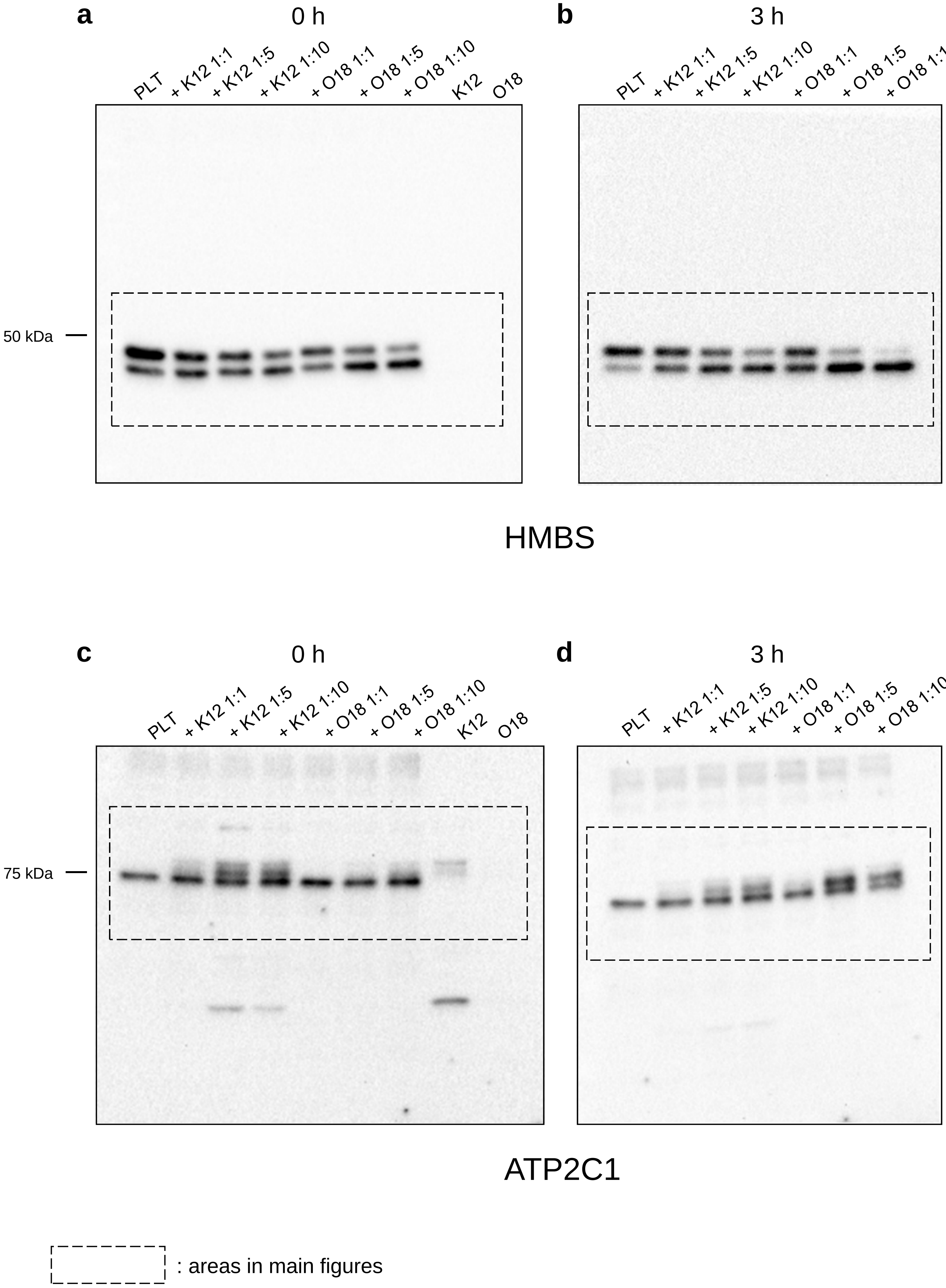

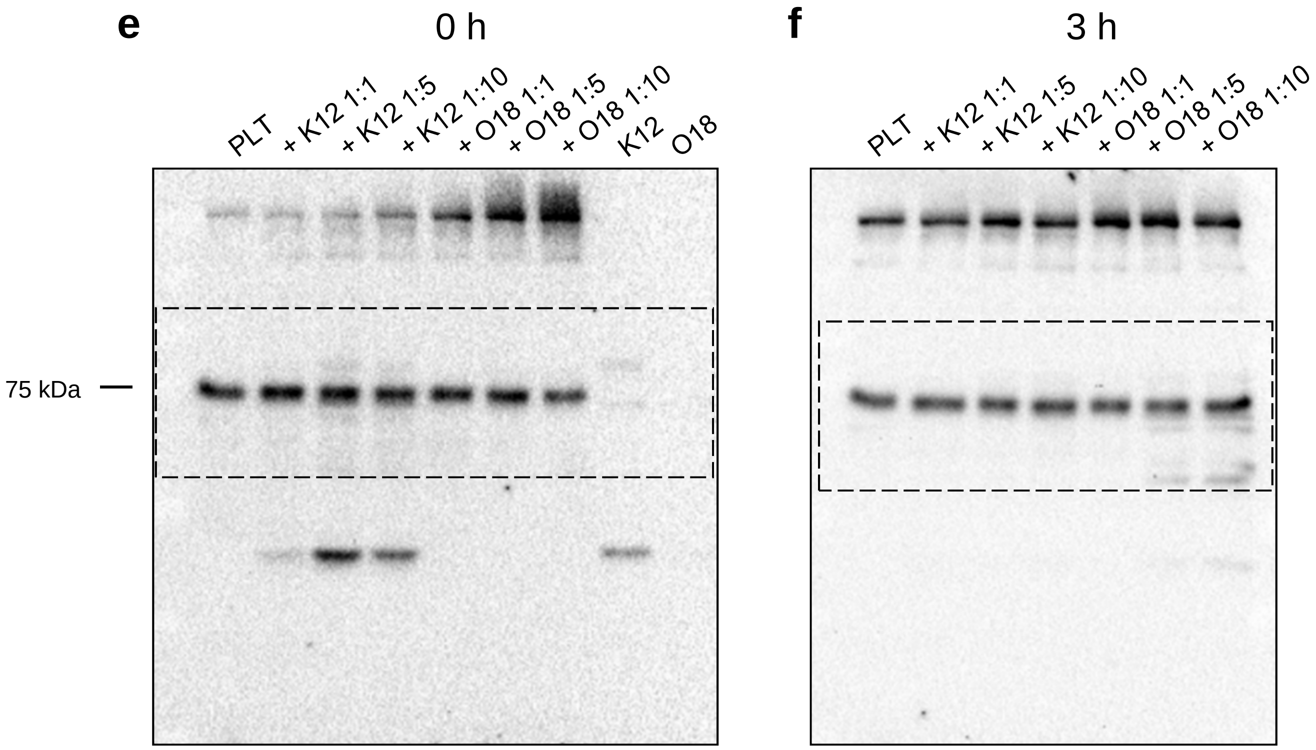

LRCH4

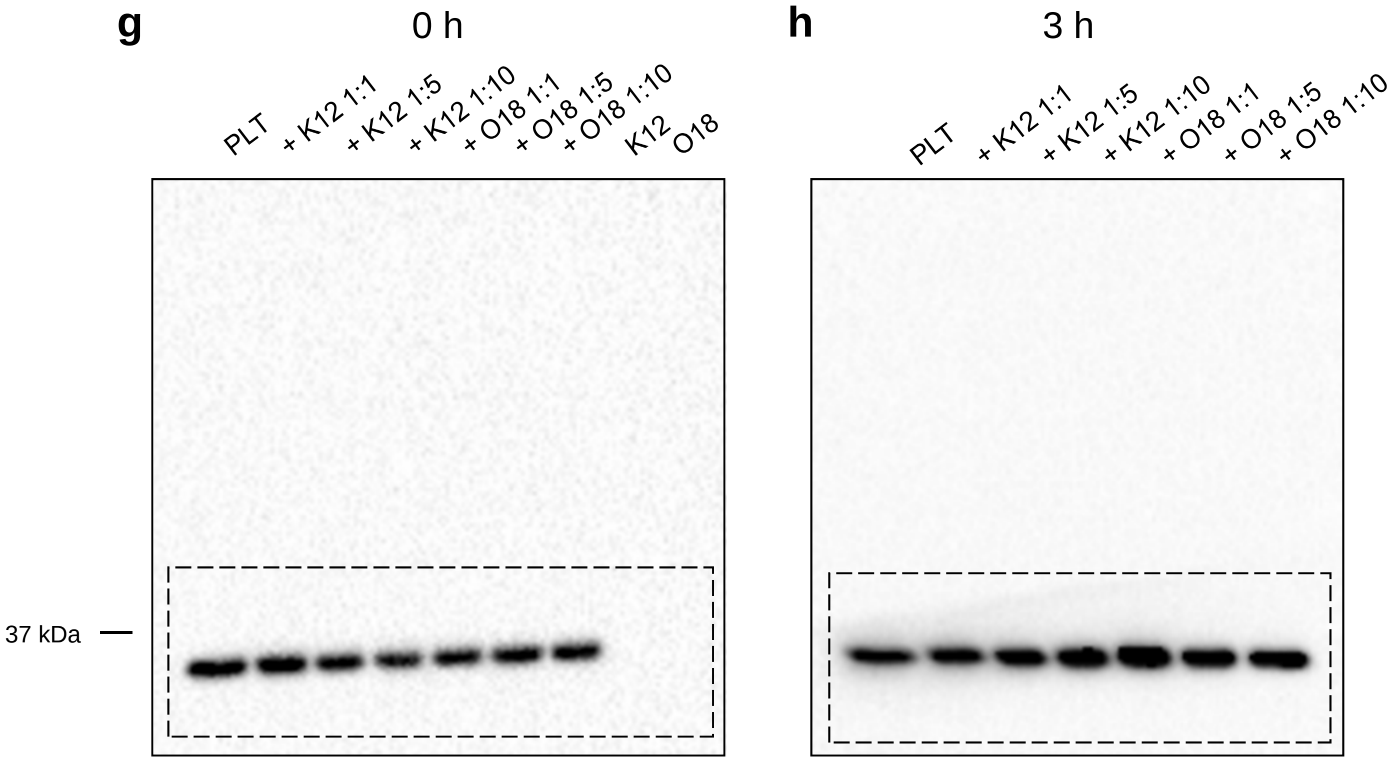

GAPDH

: areas in main figures

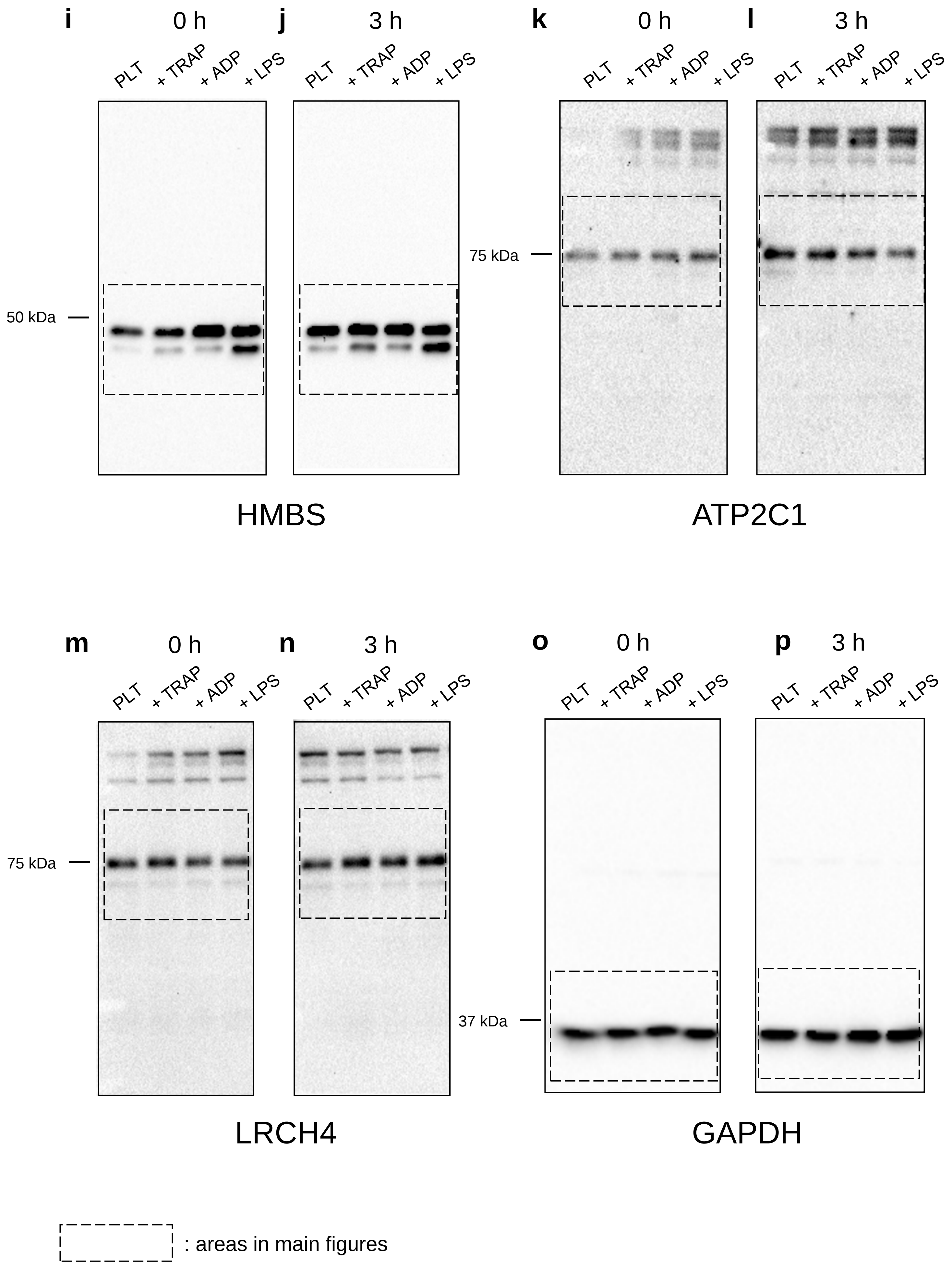

i
0 h
j
3 h
k
0 h
l
3 h
PLT
+ TRAP
+ ADP
+ LPS
50 kDa
75 kDa
HMBS
ATP2C1
m
0 h
n
3 h
o
0 h
p
3 h
75 kDa
37 kDa
LRCH4
GAPDH
: areas in main figures

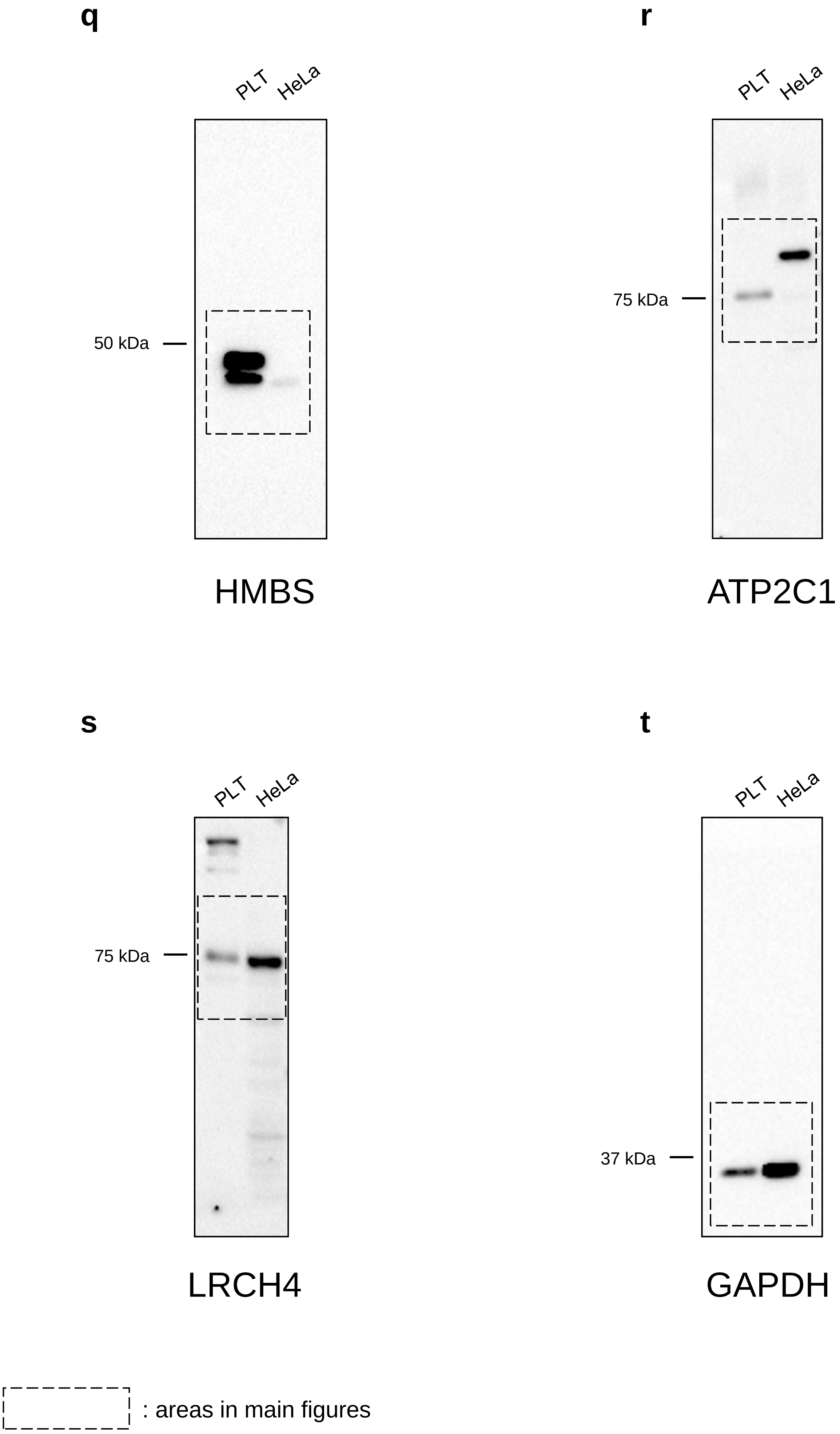
q
PLT
HeLa
50 kDa
HMBS
r
PLT
HeLa
75 kDa
ATP2C1
s
PLT
HeLa
75 kDa
LRCH4
t
PLT
HeLa
37 kDa
GAPDH
: areas in main figures

Supplementary tables

Sample description	GEO accession	Total reads	Mapped reads	Intron-spanning reads
Human platelets- control- donor1	GSM3147982	1,02E+07	7,86E+06	1,41E+06
Human platelets- control- donor2	GSM3147983	1,08E+07	7,43E+06	1,31E+06
Human platelets- control- donor3	GSM3147984	1,09E+07	7,95E+06	1,39E+06
Human platelets- control- donor4	GSM3147985	4,58E+06	2,75E+06	5,02E+05
Human platelets + E. coli K12- donor1	GSM3147986	8,85E+06	7,83E+06	1,29E+06
Human platelets + E. coli K12- donor2	GSM3147987	6,95E+06	4,84E+06	9,38E+05
Human platelets + E. coli K12- donor3	GSM3147988	9,68E+06	7,15E+06	1,36E+06
Human platelets + E. coli K12- donor4	GSM3147989	9,38E+06	5,33E+06	1,22E+06
Human platelets + E. coli O18- donor1	GSM3147990	9,09E+06	7,72E+06	1,38E+06
Human platelets + E. coli O18- donor2	GSM3147991	9,86E+06	6,89E+06	1,27E+06
Human platelets + E. coli O18- donor3	GSM3147992	9,75E+06	7,24E+06	1,32E+06
Human platelets + E. coli O18- donor4	GSM3147993	8,66E+06	5,15E+06	1,11E+06
Mean		9,06E+06	6,51E+06	1,21E+06
SD		1,76E+06	1,63E+06	2,59E+05
Max-Min		6,35E+06	5,20E+06	9,04E+05

Supplementary Table S1. Read counts of sequenced samples. For further analysis only the RNAs with high abundance (logCPM > 3) were selected.

Gene ID	Gene	Description	Co-incubation with bacteria	logFC	logCPM	PValue
ENSG00000078061	ARAF	v-raf murine sarcoma 3611 viral oncogene homolog [Source:HGNC Symbol;Acc:646]	K12	-6,33	5,17	3,48E-05
			O18	-7,05	5,21	6,74E-05
ENSG00000123240	OPTN	optineurin [Source:HGNC Symbol;Acc:17142]	K12	-5,49	5,38	2,93E-04
			O18	-4,49	5,45	7,79E-03
ENSG00000122965	RBM19	RNA binding motif protein 19 [Source:HGNC Symbol;Acc:29098]	K12	-7,26	3,94	9,91E-04
			O18	-5,58	3,97	8,41E-03
ENSG00000138629	UBL7	ubiquitin-like 7 (bone marrow stromal cell-derived) [Source:HGNC Symbol;Acc:28221]	K12	-5,26	4,65	1,87E-03
			O18	-6,59	4,66	7,15E-04
ENSG00000182473	EXOC7	exocyst complex component 7 [Source:HGNC Symbol;Acc:23214]	K12	-6,12	4,25	1,93E-03
			O18	-7,70	4,24	4,72E-04
ENSG00000113441	LNPEP	leucyl/cystinyl aminopeptidase [Source:HGNC Symbol;Acc:6656]	K12	-6,03	4,17	2,16E-03
			O18	-7,65	4,19	4,96E-04
ENSG00000077454	LRCH4	leucine-rich repeats and calponin homology (CH) domain containing 4 [Source:HGNC Symbol;Acc:6691]	K12	-4,07	4,83	4,19E-03
			O18	-8,31	4,78	4,52E-05
ENSG00000152457	DCLRE1C	DNA cross-link repair 1C [Source:HGNC Symbol;Acc:17642]	K12	-7,08	3,79	4,36E-03
			O18	-7,17	3,80	4,94E-03
ENSG00000023228	NDUFS1	NADH dehydrogenase (ubiquinone) Fe-S protein 1, 75kDa (NADH-coenzyme Q reductase) [Source:HGNC Symbol;Acc:7707]	K12	-6,11	4,52	4,99E-03
			O18	-8,06	4,56	7,69E-04
ENSG00000065675	PRKCQ	protein kinase C, theta [Source:HGNC Symbol;Acc:9410]	K12	3,53	5,96	7,95E-03
			O18	3,40	5,91	3,28E-02
ENSG00000114446	IFT57	intraflagellar transport 57 homolog (Chlamydomonas) [Source:HGNC Symbol;Acc:17367]	K12	-4,47	4,53	8,03E-03
			O18	-8,02	4,53	1,84E-04
ENSG00000133706	LARS	leucyl-tRNA synthetase [Source:HGNC Symbol;Acc:6512]	K12	-4,57	4,41	8,28E-03
			O18	-6,41	4,39	1,41E-03
ENSG00000105186	ANKRD27	ankyrin repeat domain 27 (VPS9 domain) [Source:HGNC Symbol;Acc:25310]	K12	-4,49	4,15	1,49E-02
			O18	-7,57	4,11	7,47E-04
ENSG00000198677	TTC37	tetratricopeptide repeat domain 37 [Source:HGNC Symbol;Acc:23639]	K12	-3,34	6,23	1,59E-02
			O18	-8,17	6,14	4,26E-08
ENSG00000185513	L3MBTL1	l(3)mbt-like 1 (Drosophila) [Source:HGNC Symbol;Acc:15905]	K12	-4,67	4,23	1,70E-02
			O18	-5,35	4,24	9,57E-03
ENSG00000075856	SART3	squamous cell carcinoma antigen recognized by T cells 3 [Source:HGNC Symbol;Acc:16860]	K12	-5,53	4,05	1,71E-02
			O18	-5,13	4,11	2,26E-02
ENSG00000146247	PHIP	pleckstrin homology domain interacting protein [Source:HGNC Symbol;Acc:15673]	K12	1,59	7,24	2,83E-02
			O18	-3,29	5,48	1,28E-02
ENSG00000132842	AP3B1	adaptor-related protein complex 3, beta 1 subunit [Source:HGNC Symbol;Acc:566]	K12	-3,41	4,47	2,89E-02
			O18	1,91	6,50	3,63E-02
ENSG00000197563	PIGN	phosphatidylinositol glycan anchor biosynthesis, class N [Source:HGNC Symbol;Acc:8967]	K12	4,16	6,03	3,14E-02
			O18	3,41	5,37	4,68E-02
ENSG00000143207	RFWD2	ring finger and WD repeat domain 2, E3 ubiquitin protein ligase [Source:HGNC Symbol;Acc:17440]	K12	-4,61	4,18	3,58E-02
			O18	-5,79	4,16	1,39E-02
ENSG00000149485	FADS1	fatty acid desaturase 1 [Source:HGNC Symbol;Acc:3574]	K12	-3,91	4,12	4,42E-02
			O18	-7,52	4,08	7,72E-04

Supplementary Table S2. Overlapping, significantly changed RNAs between *E. coli* K12 and O18:K1 exposed bacteria platelet profiles. The logFCs are visualized on Supplementary Figure S3.

**Annotation Cluster 1**	**Enrichment Score: 2.47**		
Category	Term	Gene Count in Category	PValue
UP_KEYWORDS	Ubl conjugation pathway	13	1,13E-04
GOTERM_BP_DIRECT	GO:0042787~protein ubiquitination involved in ubiquitin-dependent protein catabolic process	5	9,60E-03
GOTERM_MF_DIRECT	GO:0004842~ubiquitin-protein transferase activity	6	3,67E-02
**Annotation Cluster 2**	**Enrichment Score: 1.69**		
Category	Term	Gene Count in Category	PValue
UP_KEYWORDS	Golgi apparatus	11	6,11E-03
GOTERM_CC_DIRECT	GO:0005794~Golgi apparatus	11	1,54E-02
GOTERM_CC_DIRECT	GO:0000139~Golgi membrane	7	9,18E-02
**Annotation Cluster 3**	**Enrichment Score: 1.57**		
Category	Term	Gene Count in Category	PValue
GOTERM_BP_DIRECT	GO:0031145~anaphase-promoting complex-dependent catabolic process	4	9,05E-03
GOTERM_BP_DIRECT	GO:0042787~protein ubiquitination involved in ubiquitin-dependent protein catabolic process	5	9,60E-03
GOTERM_BP_DIRECT	GO:0051437~positive regulation of ubiquitin-protein ligase activity involved in regulation of mitotic cell cycle transition	3	6,40E-02
GOTERM_BP_DIRECT	GO:0043161~proteasome-mediated ubiquitin-dependent protein catabolic process	4	9,80E-02
**Annotation Cluster 4**	**Enrichment Score: 1.44**		
Category	Term	Gene Count in Category	PValue
GOTERM_MF_DIRECT	GO:0044822~poly(A) RNA binding	15	3,78E-03
GOTERM_BP_DIRECT	GO:0000398~mRNA splicing, via spliceosome	6	7,16E-03
UP_KEYWORDS	mRNA splicing	6	8,81E-03
INTERPRO	IPR012677:Nucleotide-binding, alpha-beta plait	6	1,25E-02
UP_KEYWORDS	mRNA processing	6	2,30E-02
UP_SEQ_FEATURE	domain:RRM 1	4	2,40E-02
UP_SEQ_FEATURE	domain:RRM 2	4	2,40E-02
INTERPRO	IPR000504:RNA recognition motif domain	5	3,08E-02
SMART	SM00360:RRM	5	3,94E-02
UP_KEYWORDS	RNA-binding	8	4,33E-02
GOTERM_MF_DIRECT	GO:0000166~nucleotide binding	6	4,49E-02
UP_KEYWORDS	Spliceosome	3	1,27E-01
UP_KEYWORDS	Ribonucleoprotein	4	1,74E-01
GOTERM_BP_DIRECT	GO:0008380~RNA splicing	3	2,27E-01
GOTERM_MF_DIRECT	GO:0003676~nucleic acid binding	7	4,71E-01
**Annotation Cluster 5**	**Enrichment Score: 1.33**		
Category	Term	Gene Count in Category	PValue
GOTERM_MF_DIRECT	GO:0098641~cadherin binding involved in cell-cell adhesion	6	2,30E-02
GOTERM_CC_DIRECT	GO:0005913~cell-cell adherens junction	6	2,80E-02
GOTERM_BP_DIRECT	GO:0098609~cell-cell adhesion	5	5,97E-02
GOTERM_MF_DIRECT	GO:0042802~identical protein binding	8	1,24E-01

Supplementary Table S3. DAVID gene ontology clusters in details after functional annotational analysis of *E. coli* K12 treated platelets. Clusters visualised on Fig. 3c.

Annotation Cluster 1	Enrichment Score: 4.61		
Category	Term	Gene Count in Category	PValue
UP_KEYWORDS	ATP-binding	23	5,68E-07
GOTERM_MF_DIRECT	GO:0005524~ATP binding	24	5,39E-06
UP_KEYWORDS	Nucleotide-binding	23	3,26E-05
UP_SEQ_FEATURE	nucleotide phosphate-binding region:ATP	13	3,57E-03
**Annotation Cluster 2**	**Enrichment Score: 3.66**		
Category	Term	Gene Count in Category	PValue
GOTERM_CC_DIRECT	GO:0005654~nucleoplasm	36	1,12E-07
UP_KEYWORDS	Nucleus	42	3,73E-04
GOTERM_CC_DIRECT	GO:0005634~nucleus	32	2,54E-01
**Annotation Cluster 3**	**Enrichment Score: 2.35**		
Category	Term	Gene Count in Category	PValue
GOTERM_CC_DIRECT	GO:0005765~lysosomal membrane	9	7,96E-05
GOTERM_CC_DIRECT	GO:0005764~lysosome	5	2,86E-02
UP_KEYWORDS	Lysosome	5	3,82E-02
**Annotation Cluster 4**	**Enrichment Score: 1.70**		
Category	Term	Gene Count in Category	PValue
INTERPRO	IPR017892:Protein kinase, C-terminal	4	4,92E-04
SMART	SM00133:S_TK_X	4	2,27E-03
UP_SEQ_FEATURE	domain:AGC-kinase C-terminal	4	2,67E-03
INTERPRO	IPR000961:AGC-kinase, C-terminal	4	3,05E-03
UP_SEQ_FEATURE	nucleotide phosphate-binding region:ATP	13	3,57E-03
INTERPRO	IPR008271:Serine/threonine-protein kinase, active site	7	5,63E-03
INTERPRO	IPR020454:Diacylglycerol/phorbol-ester binding	3	7,35E-03
UP_KEYWORDS	Kinase	10	8,93E-03
SMART	SM00220:S_TKc	7	1,11E-02
UP_KEYWORDS	Serine/threonine-protein kinase	7	1,18E-02
INTERPRO	IPR000719:Protein kinase, catalytic domain	8	1,32E-02
GOTERM_MF_DIRECT	GO:0004672~protein kinase activity	7	1,51E-02
GOTERM_MF_DIRECT	GO:0004674~protein serine/threonine kinase activity	7	1,85E-02
INTERPRO	IPR011009:Protein kinase-like domain	8	2,02E-02
UP_SEQ_FEATURE	binding site:ATP	8	2,05E-02
UP_SEQ_FEATURE	domain:Protein kinase	7	3,11E-02
UP_KEYWORDS	Transferase	15	3,39E-02
INTERPRO	IPR002219:Protein kinase C-like, phorbol ester/diacylglycerol binding	3	4,59E-02
SMART	SM00109:C1	3	4,59E-02
INTERPRO	IPR017441:Protein kinase, ATP binding site	6	4,78E-02
UP_SEQ_FEATURE	active site:Proton acceptor	8	4,85E-02
BIOCARTA	h_mapkPathway:MAPKinase Signaling Pathway	3	9,64E-02
GOTERM_BP_DIRECT	GO:0035556~intracellular signal transduction	5	1,75E-01
KEGG_PATHWAY	hsa04931:Insulin resistance	3	1,89E-01
GOTERM_BP_DIRECT	GO:0006468~protein phosphorylation	5	2,34E-01
GOTERM_CC_DIRECT	GO:0005622~intracellular	6	8,19E-01
**Annotation Cluster 5**	**Enrichment Score: 1.59**		
Category	Term	Gene Count in Category	PValue
GOTERM_CC_DIRECT	GO:0005794~Golgi apparatus	11	1,25E-02
GOTERM_BP_DIRECT	GO:0098609~cell-cell adhesion	6	1,59E-02
GOTERM_MF_DIRECT	GO:0098641~cadherin binding involved in cell-cell adhesion	6	2,30E-02
GOTERM_CC_DIRECT	GO:0005913~cell-cell adherens junction	6	2,49E-02
UP_KEYWORDS	Golgi apparatus	8	9,64E-02

Table continues on next page

**Annotation Cluster 6**	**Enrichment Score: 1.47**		
Category	Term	Gene Count in Category	PValue
GOTERM_CC_DIRECT	GO:0005689~U12-type spliceosomal complex	3	7,73E-03
UP_KEYWORDS	mRNA processing	6	2,21E-02
GOTERM_BP_DIRECT	GO:0000398~mRNA splicing, via spliceosome	5	3,26E-02
UP_KEYWORDS	mRNA splicing	5	3,69E-02
GOTERM_BP_DIRECT	GO:0008380~RNA splicing	4	6,14E-02
UP_KEYWORDS	Spliceosome	3	1,25E-01
**Annotation Cluster 7**	**Enrichment Score: 1.42**		
Category	Term	Gene Count in Category	PValue
GOTERM_CC_DIRECT	GO:0005794~Golgi apparatus	11	1,25E-02
GOTERM_BP_DIRECT	GO:0016192~vesicle-mediated transport	4	4,96E-02
UP_KEYWORDS	Cytoplasmic vesicle	6	8,77E-02
**Annotation Cluster 8**	**Enrichment Score: 1.40**		
Category	Term	Gene Count in Category	PValue
UP_KEYWORDS	Aminopeptidase	4	3,24E-04
COG_ONTOLOGY	Amino acid transport and metabolism	4	3,40E-03
UP_KEYWORDS	Signal-anchor	4	3,75E-01
UP_KEYWORDS	Protease	4	4,88E-01
GOTERM_BP_DIRECT	GO:0006508~proteolysis	4	5,12E-01
**Annotation Cluster 9**	**Enrichment Score: 1.31**		
Category	Term	Gene Count in Category	PValue
GOTERM_MF_DIRECT	GO:0004386~helicase activity	4	1,19E-02
UP_KEYWORDS	Helicase	4	3,18E-02
INTERPRO	IPR027417:P-loop containing nucleoside triphosphate hydrolase	7	3,03E-01
**Annotation Cluster 10**	**Enrichment Score: 1.15**		
Category	Term	Gene Count in Category	PValue
GOTERM_BP_DIRECT	GO:0006396~RNA processing	5	1,90E-03
UP_KEYWORDS	RNA-binding	8	4,14E-02
GOTERM_MF_DIRECT	GO:0000166~nucleotide binding	6	4,49E-02
INTERPRO	IPR012677:Nucleotide-binding, alpha-beta plait	5	4,82E-02
SMART	SM00360:RRM	4	1,01E-01
INTERPRO	IPR000504:RNA recognition motif domain	4	1,12E-01
UP_SEQ_FEATURE	domain:RRM 1	3	1,27E-01
UP_SEQ_FEATURE	domain:RRM 2	3	1,27E-01
UP_SEQ_FEATURE	short sequence motif:Nuclear localization signal	4	2,41E-01
GOTERM_MF_DIRECT	GO:0003676~nucleic acid binding	7	4,71E-01

Supplementary Table S4. DAVID gene ontology clusters in details after functional annotational analysis of *E. coli* O18:K1 treated platelets. Clusters visualised on Fig. S3c.
